# Pumping machine fault diagnosis based on fused RDC-RBF

**DOI:** 10.1371/journal.pone.0291777

**Published:** 2023-09-25

**Authors:** Bowen Li, S. Selvakumar Raja, Jiajun Li, Zejun Yao, Wenguang Song, Haoyuan Li

**Affiliations:** 1 School of Computer Science, Yangtze University, Jingzhou, Hubei Province, China; 2 Applied Computer Science, University of Gondar, Gonder, Amhara Region, Ethiopia; 3 China Oilfield Services Limited, Sanhe, Hebei Province, China; Mirpur University of Science and Technology, PAKISTAN

## Abstract

At present, the fault diagnosis of pumping units in major oil fields in China is time-consuming and inefficient, and there is no universal problem for high requirements of hardware resources. In this study, a fault fusion diagnosis method of pumping unit based on improved Fourier descriptor (IDF) and rapid density clustering RBF (RDC-RBF) neural network is proposed. Firstly, the minimum inertia axis of the center of gravity of the indicator diagram is obtained. The farthest point of the intersection of the inertial axis and the contour is determined as the starting point. Then Fourier transform is performed on the contour boundary of the graph to obtain the feature vector. Then, combining with the idea of fast density clustering algorithm, the number of hidden layer neurons of RBF is determined by finding the point with the highest density and using it as the hidden layer neuron. At the same time, the characteristics of Gaussian function are introduced to ensure the activity of hidden layer neurons. Finally, through dynamic adaptive cuckoo search (DACS), the step size is automatically adjusted according to the convergence speed of the objective function of RBF, and the efficiency and accuracy of RBF in different search stages are balanced. The optimal parameters such as the width and weight of RBF are determined, and the optimal RDC-RBF fault diagnosis model is established. The model is applied to the diagnosis of different fault types of pumping units, and compared with the current mainstream models. The average detection accuracy of the fusion RDC-RBF fault diagnosis method proposed in this paper reaches 96.3%. The measured results have high accuracy and short time. At the same time, this method is currently applied to oil production sites such as Shengli Oilfield in China, which greatly reduces the human resources required for fault diagnosis of pumping units in the past.

## 1 Introduction

At present, the oilfield is developing digitally, and pumping units are often used in remote field areas for oil production. Because the environment is complex and changeable and harsh, and there are many unknown factors in the underground construction, which will cause pumping unit failure. These problems can cause low oil recovery efficiency, delay project time, waste human and material resources, increase oil recovery costs, and may lead to safety accidents. Therefore, high efficiency and high precision diagnosis of pumping unit working conditions is of great significance to ensure oil recovery rate and safe production in oil fields.

Currently, the power diagram analysis method, expert system diagnosis method, computer diagnosis method, and the neural network diagnosis method, which has become popular in recent years, are Widely used domestically and internationally [1, 2]. The literature [3, 4] show that the downhole power diagram analysis method is complicated and inconvenient to operate, and the surface power diagram analysis method is too dependent on theory, so there are many limitations in practical application. T.A. Aliev [5] shows that the computerized diagnosis method still requires manual experience to determine the faults, such as pumping machine hitting the pump, which is not efficient. While the current neural network diagnosis method is gradually developed, Ying H [6] shows that it does not need to rely on manual experience, and is capable of fault diagnosis by comprehensive analysis of pumping machine working condition data with only a small consumption of resources. Li J [7] uses a multi-feature fusion model to diagnose eight types of pumping machine faults with an average accuracy of more than 93%, but the method is too demanding for oilfield. Zhou W [8] developes an intelligent oilfield fault diagnosis system using UKF-RBF model, which has good results in multi-fault classification. Hao X [9] uses iterative learning optimization BP algorithm to diagnose pumping machine well conditions with an accuracy of more than 94%, but the BP algorithm is time-consuming to train and the weights are difficult to grasp, and the experimental results are highly randomized. In recent years, with the emergence of large-scale data and the improvement of GPU computing power, the deep learning model represented by convolutional neural network (CNN) has become a research hotspot in pattern recognition, target detection and other aspects. He Y [10] and Tian H [11] use the improved CNN model, and the detection accuracy in the ideal state reached more than 95%. However, this method is not ideal when the sample of the indicator diagram is small, and the structure is too complex and the calculation is too dense. This method requires the pumping unit condition detection equipment to have large storage and running memory, and program crashes often occur in the actual application of the oil field. At the same time, there are also breakthroughs in emerging fields, such as mechanical fault diagnosis of digital twins. By using a digital twin-driven intelligent gear health management method [12] for intelligent evaluation of pumping unit parts, and using a new gear fatigue monitoring index [13] to predict the life of pumping unit parts is promising in the future. At present, the intelligent intermittent fault diagnosis, and prediction method of steer-by-wire (SBW) system based on composite degradation model [14] and the event-triggered discrete component prediction of hybrid system based on degradation model selection [15] also have good prospects in the field of pumping unit diagnosis. Wang [16] proposes an improved one-dimensional ternary modeling approach to compute the parameters of a one-dimensional ternary model that is usually chosen by trial and error through shape transformation. This method also has a significant effect in the application of fault feature extraction. At the same time, the artificial ant colony algorithm proposed by the author can also quickly obtain the optimal solution of the algorithm. Hidayat [17] proposes a hybrid feature selection technique composed of Pearson correlation coefficient and random forest model, which also has important reference significance in fault feature extraction of pumping units.

The advantage of radial basis function (RBF) neural network is that it can independently seek data centers. Its structure is simpler than BP, CNN and so on. Theoretically, it is the network with the strongest mapping ability, and the classification effect is better. At the same time, it has low hardware requirements and less burden on application scenarios, and is widely used in industrial fields such as fault diagnosis and pattern recognition. The problem of RBF construction lies in the structure design. In the early days, empirical trial-and-error methods were used to establish the structure of the hidden layers, but it was difficult to obtain a compact network structure with guaranteed accuracy. Clustering algorithms are often used to determine the structure of RBF neural networks, such as K-Means, Fuzzy C-Means (FCM), etc. These algorithms can determine the radial action range of the hidden layer neurons based on the distance between the clustering centers, but they cannot determine the number of hidden layer neurons, which will affect the generalization ability of the neural network. Meanwhile, for improving the training speed of RBF and the problem that RBF is easy to fall into local optimum, better results can be achieved by optimizing RBF. Nowadays, the more widely used ones are particle swarm optimization (PSO), genetic algorithm (GA) and cuckoo search (CS). The literature [18, 19] show that PSO is prone to early maturity, GA is operationally complex, and CS is more robust and superiority-seeking in multi-peak optimization. However, CS is prone to the deficiency of oscillation around the global optimum as well as lack of dynamism in the late iteration. At the same time, in the data preprocessing part, the Fourier descriptor has the invariance of scale, translation and rotation [20, 21], and has excellent description ability for the single closed curve characteristics, such as the dynamometer diagram of the pumping unit working condition. Combining the properties of traditional Fourier descriptors, when the Fourier descriptors are normalized, the phase information will be eliminated, so the Fourier descriptors will be invariant. However, the phase information of the image is very important for accurately retrieving the shape features of the object. Therefore, although the traditional Fourier descriptors have advantages, they also reduce the accuracy of image shape retrieval. In order to use the phase information in the Fourier coefficients in the shape retrieval of the indicator diagram, it is necessary to ensure that the invariance is obtained when the edge starting point and the edge direction change. Therefore, when performing indicator diagram retrieval, the most important thing is to determine the edge starting point of the image.

Although the current domestic and foreign methods have their significant advantages and have made outstanding contributions to the fault diagnosis of pumping units, there are still some shortcomings. Therefore, based on the above situation, it is proposed to determine the starting point position of the indicator diagram contour through the minimum inertia axis, and then perform Fourier transform on the contour boundary of the graph to obtain the feature vector. This method makes the feature extraction of the indicator diagram achieve the best matching between the contours and the effect of the starting point independence, and reduces the influence of the indicator diagram change on the accuracy. Then, the good clustering characteristics of the improved fast density clustering algorithm are used in the structural design of RBF neural network. By finding the point with the highest density and using it as the hidden layer neuron, the number of hidden layer neurons is determined. And the characteristics of Gaussian function are introduced to ensure the activity of each hidden layer neuron. On the premise of ensuring the activity of neurons, this method obtains RBF neural network with more compact structure and better generalization ability by finding points with larger local density values. Finally, the dynamic discovery probability and adaptive step size are introduced to solve the shortcomings of CS, and RBF is optimized to balance the efficiency and accuracy of RBF in different search stages and accelerate the convergence speed. And the optimal RDC-RBF model is constructed to diagnose the fault of pumping unit. The experimental results show that the proposed method has certain advantages.

## 2 Improved Fourier descriptor

Fourier descriptors have the characteristics of translation, rotation and scaling invariance. However, it is easy to be affected by the position of the starting point when performing image retrieval and feature extraction on the indicator diagram. In view of this situation, this paper proposes a method to determine the position of the starting point of the dynamometer contour by the minimum inertia axis, and then carries out the boundary Fourier transform. In rigid body mechanics, if the mass of a rigid body *M* is uniformly distributed and axisymmetric, it has the smallest moment of inertia when it is rotated around the axis of symmetry [22, 23]. Introducing this idea, in the grayscale diagram of the power diagram, the training sets targets are composed of the set of P_*pixel*_. The *N* pixel points are considered as mass microelements, so the moments of inertia of *N* mass microelements are calculated by Eq (2) and summed up by integration to finally obtain the linear moment of inertia of this schematic power map.

$$\begin{array}{c} {\text{P}}_{pixel}=\left\{\left(x_{i},y_{i}\right)|i\in \left[1,N\right]\right\} \end{array}$$ (1)

$$\begin{array}{c} d_{i}=\frac{|y-(x-x_{c})\text{tan}\;\theta -y_{c}|}{\sqrt{1+\text{tan}\;\theta^{2}}} \end{array}$$ (2)

$$\begin{array}{c} I=\underset{i=1}{\sum^{N}}\;d_{i}^{2} \end{array}$$ (3)

**Fig 1:**
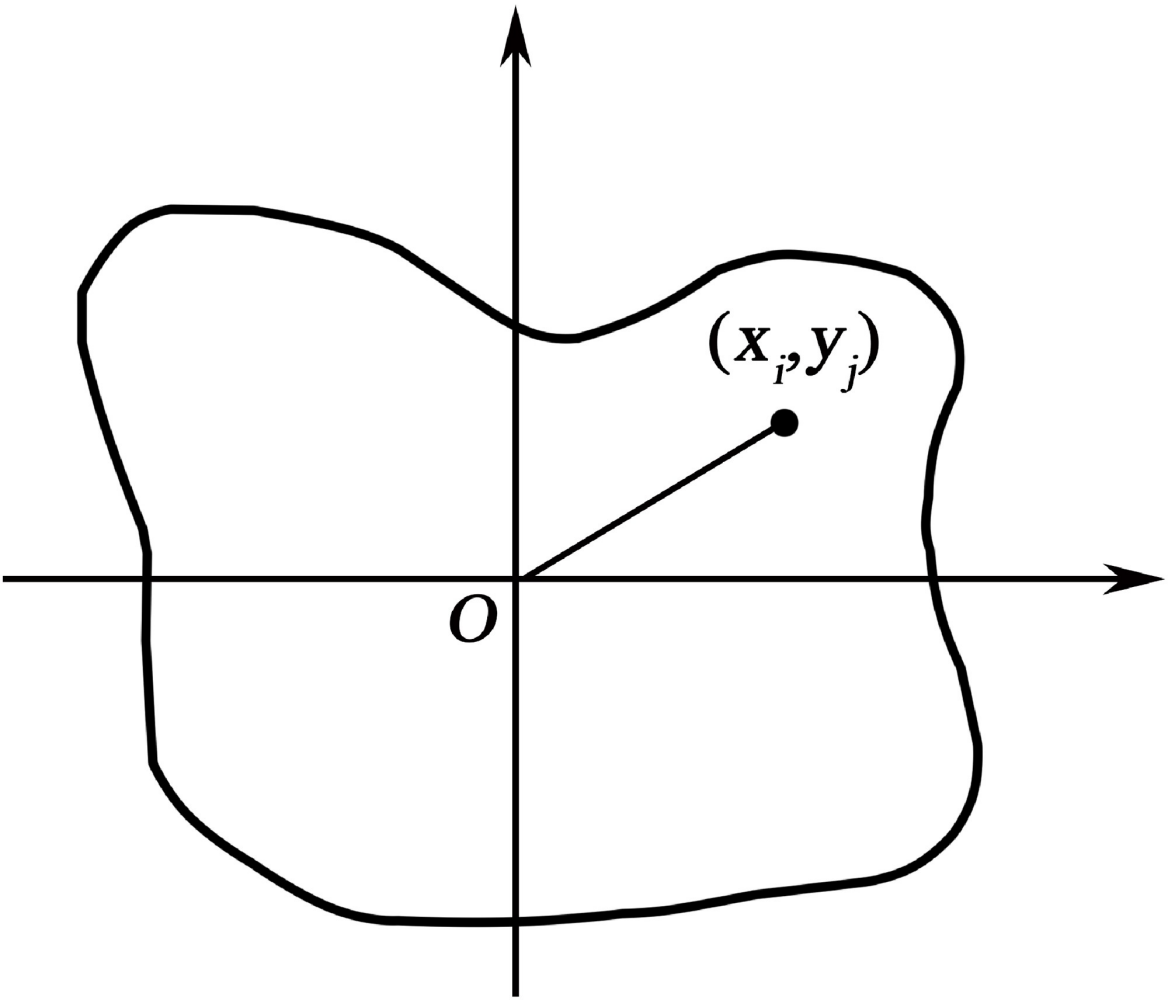
Graphical centre of gravity.

In Eq (1), *N* is the total number of pixels, and (*x*_*i*_, *y*_*i*_) is the coordinates of the established starting point. In Eq (2), (*x*_*c*_, *y*_*c*_) is the barycenter coordinate of the image, *θ* is the intersection of the center of gravity and the outline of the figure corresponding to the *N* pixels that make up the work diagram, and the angle determined by the two-dimensional coordinate axis. The coordinates of the center of gravity are shown in Fig 1. The formula for calculating the center of gravity of the work diagram is shown below.

$$\begin{array}{c} x_{c}=\frac{\sum p_{i}x_{i}}{\sum p_{i}} \end{array}$$ (4)

$$\begin{array}{c} y_{c}=\frac{\sum p_{i}y_{i}}{\sum p_{i}} \end{array}$$ (5)

In Eqs (4) and (5), *p*_*i*_ is the pixel value of the starting point.

Since the principal direction of the original normalized shape is horizontal, the angle between the obtained minimum inertia axis and the coordinate axis is defined as the principal direction *φ* in the new normalized shape of the oscillogram by the Fourier descriptor. It is inferred from Eq (7) that the estimated boundary from the principal direction of the shape, by shifting the amount of phase influence *P*_*phase*_ of the arc length, can then be eliminated when the information about the shape and position of the oscillogram changes the starting point phase influence. In the Eq (7), *a* is the position transformation of the starting point of the image, *β* is the scaling amplitude, *K* is the number of points that constitute the boundary of the image, and *j* is the imaginary number.

$$\begin{array}{c} P_{phase}=e^{-j\frac{2\pi }{K}a} \end{array}$$ (6)

$$\begin{array}{c} \frac{s^{\prime }\left(1\right)}{\left|\right|s^{\prime }\left(1\right)\left|\right|}=\frac{\beta e^{j\varphi }e^{-j\frac{2\pi }{K}a}s\left(1\right)}{\beta e^{j\varphi }e^{-j\frac{2\pi }{K}a}s\left(1\right)}=e^{j\varphi }e^{-j\frac{2\pi }{K}a} \end{array}$$ (7)

The new improved normalized Fourier descriptor *z*′(*k*) is finally obtained, as shown in Eq (8) below, *N* is the number of sampling points on the image contour.

$$\begin{array}{c} z^{\prime }\left(k\right)=\frac{s^{\prime }\left(k\right)e^{j\theta }}{∥s(1)∥},k=0,1,2,\cdots ,N-1 \end{array}$$ (8)

**Fig 2:**
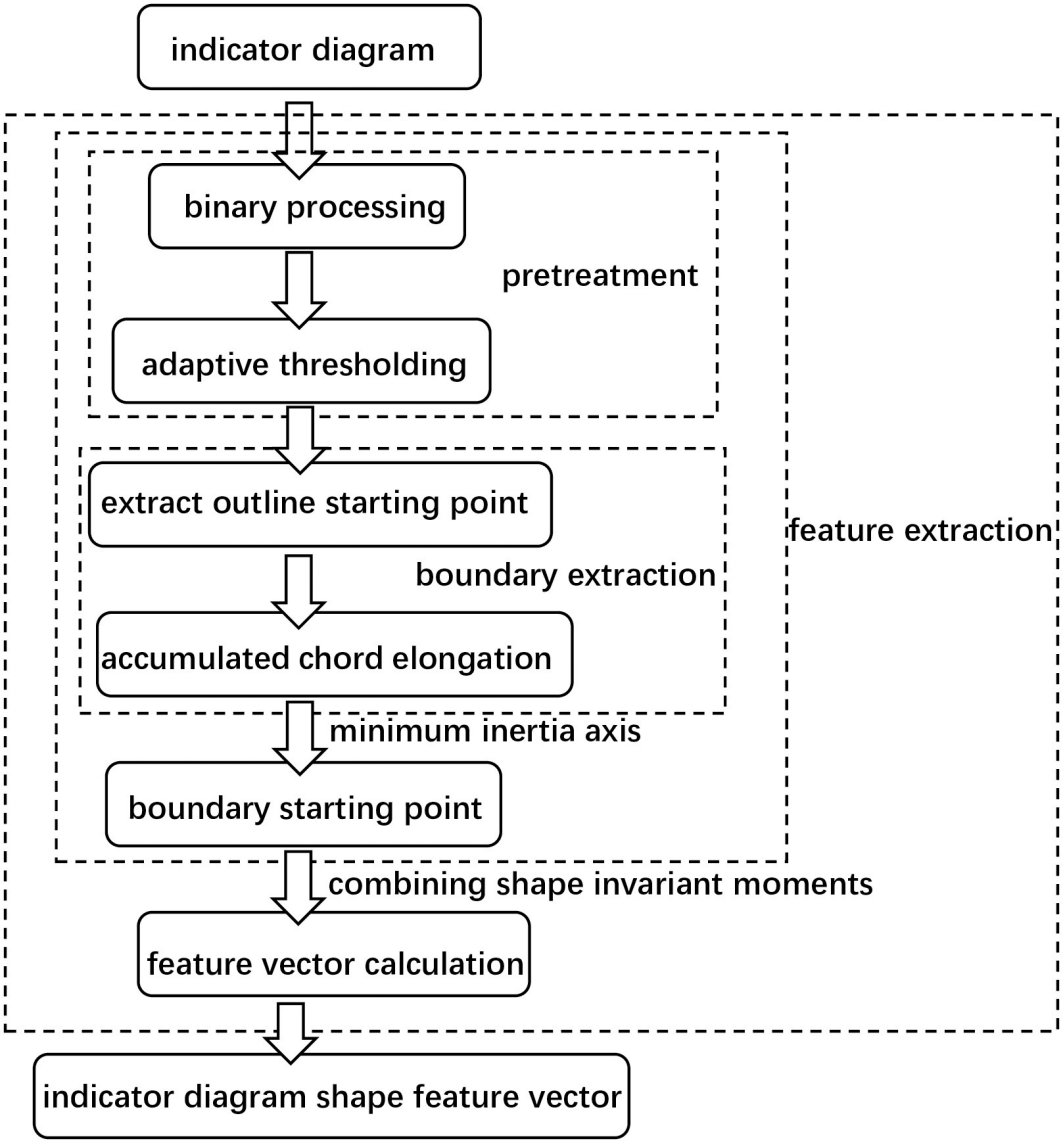
framework for extracting feature vectors of a power diagram.

The improved Fourier descriptor(IDF) is applied to the pre-processing of the schematic graph data of the pumping machine working conditions. Firstly, the coordinate points of the contour edge of the image are extracted and the extracted coordinates are differenced, and then the contour coordinates of the schematic graph with equal spacing are obtained. Then the center of gravity of the graph is calculated, and the farthest point of the intersection of the minimum inertia axis of the center of gravity and the outline of the schematic figure is determined as the starting point, and then the Fourier transform of the outline boundary of the graph is done to obtain the feature vector required for the experiment. The advantage is that the best starting point is selected to improve the similarity between classes, and the best matching between contours is achieved after comparing the schematic diagram from multiple angles, and the starting point is irrelevant. This approach also reduces the influence of the schematic transformation on the results, and improves the stability and accuracy of image recognition. The framework of extracting the pumping machine working conditions by improving the Fourier descriptor combined with the shape invariant moment is shown in Fig 2.

## 3 Pumping machine fault diagnosis model design

### 3.1 RBF neural network

**Fig 3:**
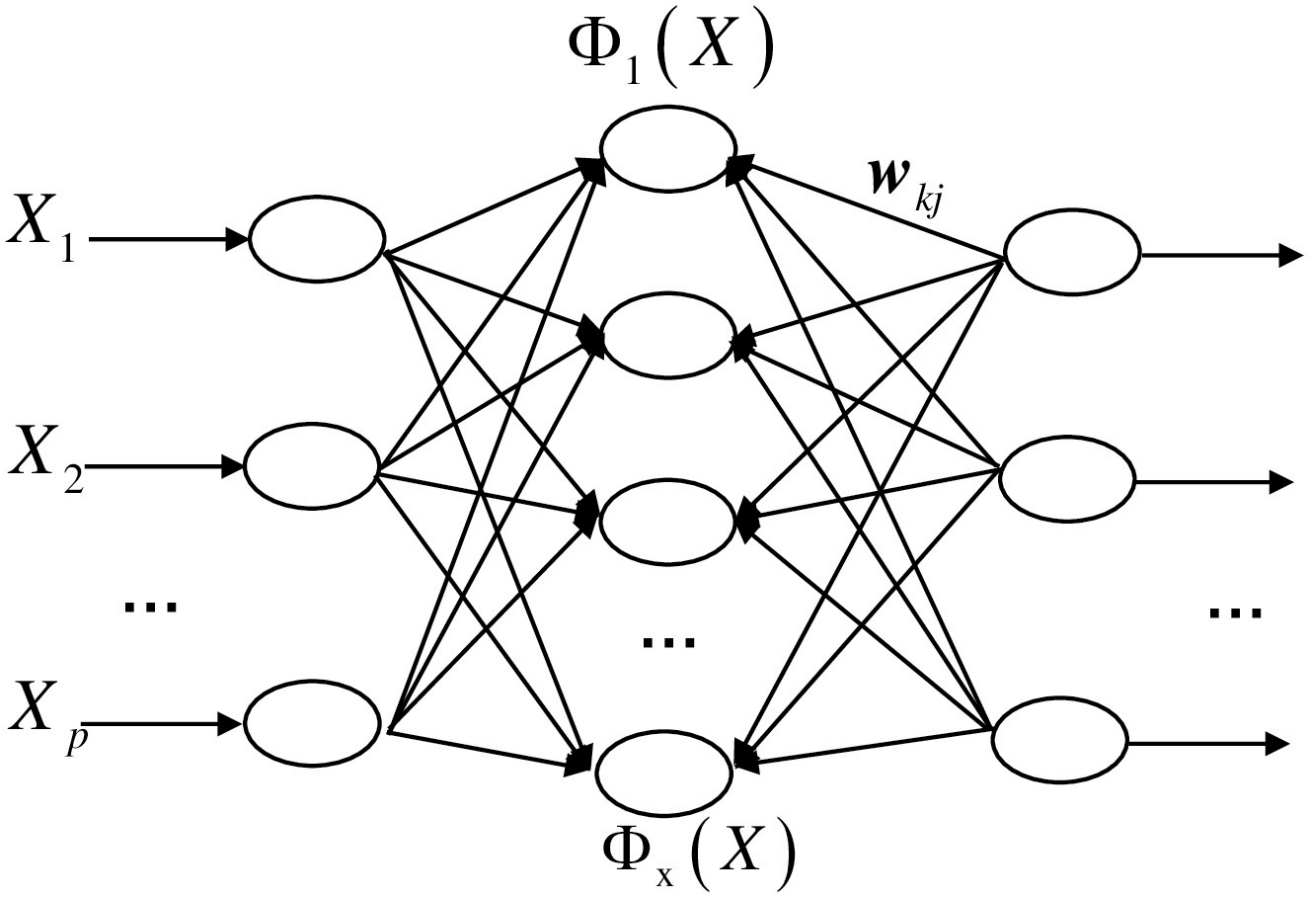
RBF neural network structure diagram.

The overall structure of the RBF neural network is shown in Fig 3 and consists of three parts: the network input layer, the network implicit layer, and the network output layer [24].

Gaussian functions are commonly used as activation functions for neurons in the hidden layer [25, 26].

$$\begin{array}{c} R(x_{p}-c_{i})=\text{exp}\;(-\frac{1}{2\sigma^{2}}\left|\right|x_{p}-c_{i}|{|}^{2}) \end{array}$$ (9)

In Eq (9), *σ* is the width of the neuron in the hidden layer, *c* is the central node, and |*x*_*p*_ − *c*_*i*_|| is the distance from the sample point to the centroid. The output of the RBF is shown below.

$$\begin{array}{c} y_{j}=\sum_{i=1}^{h}w_{ij}\;\text{exp}\;(-\frac{1}{2\sigma^{2}}{∥x_{p}-c_{i}∥}^{2}) \end{array}$$ (10)

In Eq (10), *w* is the connection weight from the hidden layer to the output layer. Thus the effectiveness of the RBF neural network model is influenced by the centroid *c*. The width *σ* and the connection weights *w*, and the design of RBF neural network includes two parts: structure construction and parameter training. In the paper, an improved rapid density clustering algorithm is used to determine the initial structure of the network, while the target parameters are optimized by an improved CS algorithm.

### 3.2 Rapid density clustering algorithm

Clustering analysis divides samples into several categories based on similarity, but it must determine the number of categories in advance. At the same time, the method must go through a lot of iterations to find the optimal clustering results, which increases the time burden. The core idea of the rapid density clustering algorithm is that the centre of the cluster is tightly surrounded by other points of lower density and away from other points of higher density. In the process of finding the cluster centre, for any data point *i*, two values are calculated: the local density value *ρ*_*i*_ of each point and the minimum distance *δ*_*i*_ from the point to the other denser points. The formula for calculating the local density value of data point *i*, and the formula for calculating the minimum distance *δ*_*i*_ from the data point *i* to the other denser points are Eqs (11) and (12) respectively.

$$\begin{array}{c} \rho_{i}=\sum_{j}\chi (d_{ij}-d_{c}) \end{array}$$ (11)

$$\begin{array}{c} \delta_{i}=\underset{j:\rho_{j}>\rho_{i}}{\min}\left(d_{ij}\right) \end{array}$$ (12)

When *x* < 0,*χ*(*x*) = 1; when *x* ≥ 0, *χ*(*x*) = 0. *d*_*c*_ is the premise parameter and F denotes the truncation distance. The points with the maximum value of *ρ* and the minimum value of *δ* are taken as the clustering centres. The non-central data samples are sequentially assigned to the class in which the nearest cluster centre with a higher density value is located. This clustering process has problems with online clustering as the entire data sample needs to be obtained in advance before clustering. Also the final clustering effect is influenced by the truncation distance *d*_*c*_. Therefore, this paper combines the properties of Gaussian functions to improve the clustering and apply it to the structural design of RBF neural networks.

### 3.3 Design of RBF network structure based on rapid density clustering algorithm

Based on the idea of rapid density clustering algorithm, the structure of the RBF neural network is determined by finding the points with higher local density values as the centres of neurons in the hidden layer. To address the above shortcomings of the rapid density clustering algorithm, the following improvements are made.

The activation function of RBF neurons is a Gaussian function, and the evaluation index of hidden layer neuron activity is Eq (13)

$$\begin{array}{c} {\text{AC}}_{ij}=e^{e^{-{∥x_{i}-c_{j}∥}^{2}/\sigma_{j}^{2}}}⩾V \end{array}$$ (13)

AC_*ij*_ is the activity of the *j*th hidden layer neuron after being activated by the *i*th sample, the higher the value of AC, the stronger the activity of the neuron; *V* is the threshold of neuron activity to ensure that the activity of the hidden layer neuron is large enough. The input vector, the central vector and the radial action range need to satisfy the following relations.

$$\begin{array}{c} \frac{∥x_{i}-c_{j}∥}{\sigma_{i}}⩽\sqrt{∥\text{ln}V∥} \end{array}$$ (14)

That is the distance between the input vector and the centre vector of the neuron in the hidden layer needs to satisfy the following relationship:

$$\begin{array}{c} ∥x_{i}-c_{j}∥⩽\sigma_{j}\sqrt{∥\text{ln}V∥} \end{array}$$ (15)

where the neuronal activity threshold A is taken according to the experiment. Therefore, the truncation distance is related to the radial range of action of the neuron and the activity of the neuron. In order to achieve online clustering, in this paper, the neural network structure is determined by sequentially adjusting the structure of the training samples into the neural network: adding a hidden layer neuron or adjusting the existing hidden layer neuron.

The main idea behind the design of the hidden layer structure is to determine whether the current sample can be guaranteed to be active enough when activating its nearest hidden layer neuron. If the activity can be guaranteed, the neuron can be classified into the class where the current hidden layer neuron is located, and vice versa; Secondly, by density comparison, the point with higher density will be used as the hidden layer neuron. Thus the design of RBF neural network structure based on rapid density clustering in the paper can be divided into two cases: neuron growth mechanism and neuron regulation mechanism.

#### Neuron growth mechanism

The first data sample is taken as the centre of the first hidden layer neuron, and the corresponding radial action range and output weights are set.

$$\begin{array}{c} c_{1}=x_{1} \end{array}$$ (16)

$$\begin{array}{c} \sigma_{1}=1 \end{array}$$ (17)

$$\begin{array}{c} w_{1}=y_{d1} \end{array}$$ (18)

At the moment *k*, assuming that there are already *j* hidden layer neurons. And when the *k*th data sample enters the network, find the nearest hidden layer neuron *k*_min_ to the stingy current sample.

$$\begin{array}{c} k_{\text{min}}=\text{arg}\min_{h\in [1,j]}\left\{\text{dist}\left(x_{k},c_{h}\right)\right\} \end{array}$$ (19)

$$\begin{array}{c} D=\text{dist}(x_{k},c_{k_{\text{min}}}) \end{array}$$ (20)

This distance veil is compared with the radial action paradigm of the neuron in the hidden layer. If $D⩽\sigma_{k_{\text{min}}}\sqrt{∥\text{ln}V∥}$, the current sample is not considered to guarantee the activity of the neuron, and the *k*th sample is used as the centre of the new neuron, with its radial action parametrization and output weights set.

$$\begin{array}{c} c_{j+1}=x_{k} \end{array}$$ (21)

$$\begin{array}{c} \sigma_{j+1}=1 \end{array}$$ (22)

$$\begin{array}{c} w_{j+1}=y_{dk} \end{array}$$ (23)

#### Neuron adjustment mechanism

At the moment *k*, if $D⩽\sigma_{k_{\text{min}}}\sqrt{∥\text{ln}V∥}$, the current network is considered to be able to learn the new sample. Comparing the local density value of the current sample with that hidden layer neuron, and selecting the point with the greater density value as the new hidden layer neuron. The local density of data point *i* is calculated by the formula.

$$\begin{array}{c} P_{i}=\sum_{c_{i}\neq x_{j}}\;\text{exp}\;(-{∥c_{i}-x_{j}∥}^{2}/d_{i}^{2}) \end{array}$$ (24)

The *x*_*j*_ is the sample points included in the action paradigm of *c*_*i*_; *d*_*i*_ is the local action paradigm of the number of hanging points. From Eq (24), it can be seen that if the local density of a data point is larger, it means that more sample points are clustered around that point; if the density of the hidden layer neuron is larger, it means that more samples are activated by that neuron. The local density value of the current input sample point *k* is compared with the local density value of the hidden layer neuron *k*_min_. If $P_{k}>P_{k_{\text{min}}}$, when the input sample replaces the existing hidden layer neuron, it becomes a new hidden layer neuron, and the initial parameters are set as flow:

$$\begin{array}{c} c_{k_{\text{min}}}=x_{k} \end{array}$$ (25)

$$\begin{array}{c} w_{k_{\text{min}}}=\frac{n_{k_{\text{min}}}w_{k_{\text{min}}}+y_{dj}}{n_{k_{\text{min}}}+1} \end{array}$$ (26)

$$\begin{array}{c} \sigma_{k_{\text{min}}}=\max\left(dist\left(c_{k_{\text{min}}},x_{k_{\text{min}}}\right)\right) \end{array}$$ (27)

The *k*_min_ denotes all samples activated by the *k*th hidden layer neuron; $n_{k_{\text{min}}}$ denotes the number of samples activated. If $P_{j}\leq P_{k_{\text{min}}}$, the existing hidden layer neuron remains unchanged and only the radial range of action of the neuron and the connection weights to the output layer need to be adjusted in Eqs (26) and (27)).

#### 3.4 RDC-RBF neural network design steps

**Fig 4:**
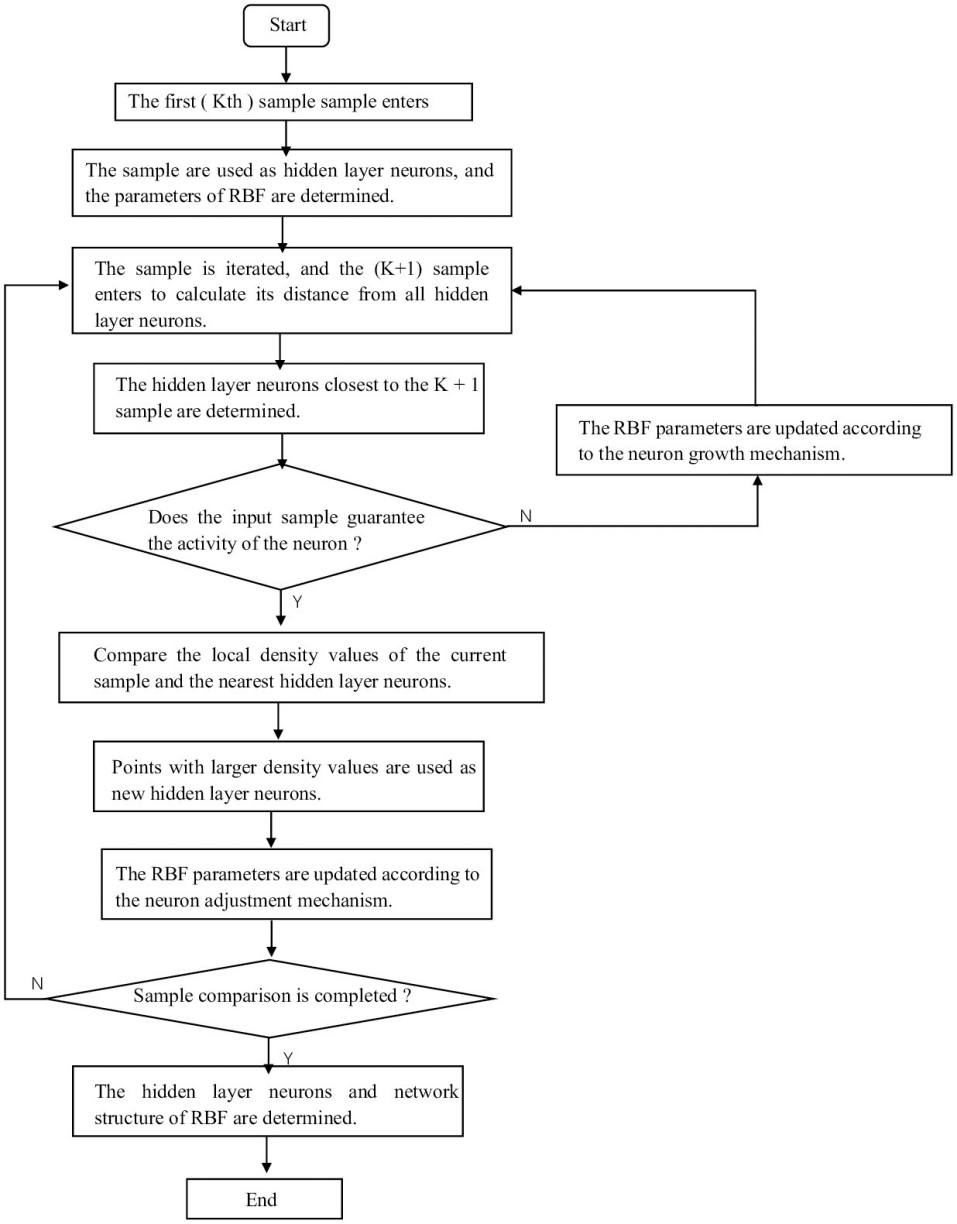
Flowchart of RDC-RBF.

The steps of RDC-RBF are as follows, and the flow chart is shown in Fig 4.

1. At the initial moment, the number of hidden layer neurons is 0.
2. When the first sample enters the network, it is treated as the first hidden layer neuron and its centre, radial range of action and connection weights are set according to Eqs (16)–(18)
3. When the *k*th sample is fed into the network, its distance from all the current hidden layer neurons is calculated, and the hidden layer neuron with the closest distance to the *k*th sample is found *k*_min_.
4. Determining whether the input sample can guarantee the activity of the *k*_min_th neuron. If not, adding a new hidden layer neuron to the network and assign the initial parameters according to Eqs (21)–(23), then turn to Eq (14); Otherwise, perform the next step.
5. If the neuron activity is guaranteed, the local density values of the current sample and the nearest hidden layer neuron are compared. The point with the higher density value is selected as the new hidden layer neuron, and the centre, radial range of action and weights are updated according to Eqs (25)–(27), then turn to Eq (14).
6. If all samples are compared, the neural network structure is determined; Otherwise, *k* = *k* + 1, turn to Eq (14).

The algorithm uses the idea of rapid density clustering in the design of the RBF network structure, and combines it with the properties of Gaussian functions to make a compact structure. At the same time, the better initial parameters set in the structure construction process can also improve the convergence speed of the network.

## 4 Optimization of pumping unit fault diagnosis model

### 4.1 Dynamic adaptive cuckoo search algorithm

The advantages of the cuckoo search algorithm are the few parameters required, excellent search paths and powerful merit-seeking ability [27], but it also suffers from slow convergence and lack of dynamism in the later stages. To remedy the shortcomings of CS and improve its performance, this paper introduces dynamic discovery probability and adaptive step size factor to optimize it.

The CS algorithm seeks the optimal solution by simulating the cuckoo in nature and by using the nests of other birds to parasitize the eggs’ habits [28, 29]. Each cuckoo is set to lay one egg at a time and then find a nest to place, and the process is randomly chosen. The overall number of nests is fixed unchanged, and the cuckoo selected the most superior nest from those found in each round to keep for the next generation. The probability that a host parasitized by a cuckoo will find an abnormal egg is set to *p*_*a*_, *p*_*a*_ ∈ [0, 1]. Based on the above rules, the cuckoo will search for the host’s nest location by Levy flight.

$$\begin{array}{c} x_{i}^{\left(t+1\right)}=x_{i}^{\left(t\right)}+\alpha ⊕L\left(\lambda \right) \end{array}$$ (28)

In Eq (28), $x_{i}^{\left(t\right)}$ is the position of the *i*th nest in the *t*th iteration, *α* is the step size factor, and *L*(λ) denotes the Levy random search path:

$$\begin{array}{c} L\left(\lambda \right)=\frac{u}{100|v{|}^{1/\beta }}(x_{i}^{\left(t\right)}-x_{b}^{\left(t\right)}) \end{array}$$ (29)

In Eq (29), *u* ∼ *N*(0, *σ*^2^), *v* ∼ *N*(0, 1), $x_{b}^{\left(t\right)}$ denotes the position of the best bird nest in the tth iteration. After the bird’s nest position is updated, a random number *r* ∈ [0, 1] is generated. when *r* > *p*_*a*_, the bird’s nest position is updated randomly again.

By analyzing the CS algorithm as a whole, it can be found that when the discovery probability *p*_*a*_ is kept constant, the bird nests are replaced with equal probability regardless of their merits, which causes a certain loss of merit seeking. Meanwhile, *L*(λ) is influenced by *u* and *v*, which leads to a strong randomness of step size. If a large step length is used throughout the process, the result will fall into a local optimum, while a small step length throughout the process will undoubtedly sacrifice the search efficiency, although it can make the result better. Therefore, a dynamic discovery probability *p*_*d*_ and an adaptive step size *s* can be introduced to make the better nests easily retained and to keep a balance between search efficiency and accuracy in different search stages to achieve an optimal result. The probability of dynamic discovery is:

$$\begin{array}{c} p_{d}=\frac{p_{\max}-p_{\text{min}}}{g-1}\times (g_{\max}-1) \end{array}$$ (30)

In Eq (30), *p*_max_ and *p*_min_ denote the maximum and minimum discovery probabilities respectively, and *g*_max_ and *g* denote the initial maximum and current number of CS iterations respectively.

The size of step *s* is adaptively adjusted according to the distance of the current search value from the global optimum, where the step size of the (*t* + 1)th generation search path is

$$\begin{array}{c} s_{i}^{t+1}=|1-f^{\prime }/f|s_{i}^{t} \end{array}$$ (31)

In Eq (31), *f* denotes the objective function value of the current best nest, *f*^′^ denotes the objective function value corresponding to the position of the merit-selected nest after the next round of update, and $s_{i}^{t}$ is the step size of the search path in the *t*th generation. Therefore, the optimized DACS algorithm can be obtained:

$$\begin{array}{c} x_{i}^{t+1}=x_{i}^{t}+|1-f^{\prime }|f|\alpha ⊕L\left(\lambda \right) \end{array}$$ (32)

It can be derived from the formula that when the objective function converges quickly, |1 − *f*^′^/*f*| increases correspondingly, so that CS maintains a large step size for the optimization search; on the contrary, when the objective function converges slowly, the search step size also decreases accordingly to avoid missing the global optimum. Therefore, DACS can maximize the elimination of inferior results compared with CS, and substantially improve the efficiency of the search.

### 4.2 DACS optimizes RBF algorithm process

By introducing dynamic discovery probability and adaptive step size, the improved DACS algorithm optimizes the RDC-RBF neural network as follows:

1. Initialize the parameters of CS including the total number of nests n, the objective function *f* (*x*), and the maximum number of iterations *g*_max_, etc. Introducing dynamic discovery probability and adaptive step size. According to the three-layer structure of RBF, the initial positions of *n* bird nests are determined, representing the parameters that need to be optimized for the RBF neural network such as the centroid *c*, the width *σ*, and the connection weights *w*.
2. Calculate the objective function of the current initialized nest, and then select the smallest objective function value marked as *f* from it, and record the position corresponding to the nest as *x*.
3. The adaptive step introduced by 1 is used to dynamically adjust the speed of iteration, and the nest positions are updated in one round. The objective function of this group of nests is calculated again, and the smallest objective function is selected and compared with *f*, and the better value is assigned to *f*^′^.
4. At this point a set of random numbers *r* is generated, where *r* ∈ [0, 1]. *r* and *P*_*d*_ are compared in terms of size, and when *r* > *P*_d_ appears, the nest position is updated again. The objective function value is calculated, and the smallest one is selected as *f*^′^. Its corresponding position *x*^′^ is recorded. Compare *f*^′^ with *f*, select the minimum value to assign to *f* and assign its nest location to *x* When *r* < *P*_*d*_, keep the nest location in 3 without updating, directly compare *f*^′^ with *f* and assign the merit to *f* and record the corresponding nest location.
5. By cyclic update iterations, when the objective function value meets the requirements and reaches the maximum number of iterations *g*_max_, the optimal objective function *f* will be obtained and its corresponding bird nest location *x* will be decoded as the parameters such as the centroid *c*, width *σ* and connection weights *w* of the RBF neural network.

**Fig 5:**
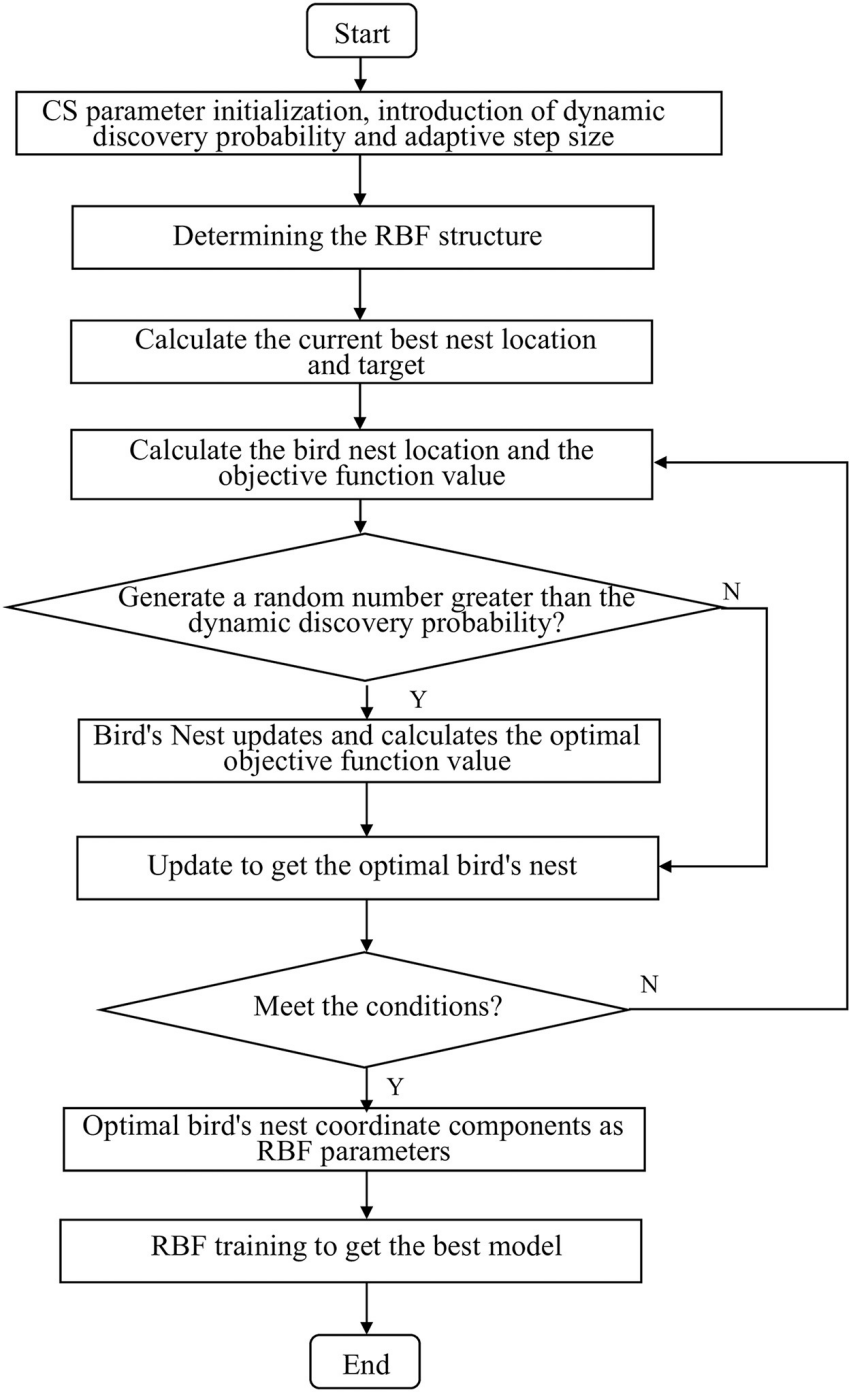
DACS optimized RBF flow chart.

The flow chart for optimizing RBF using DACS is shown in Fig 5:

## 5 Experimental data analysis and validation

### 5.1 Failure type analysis

**Fig 6:**
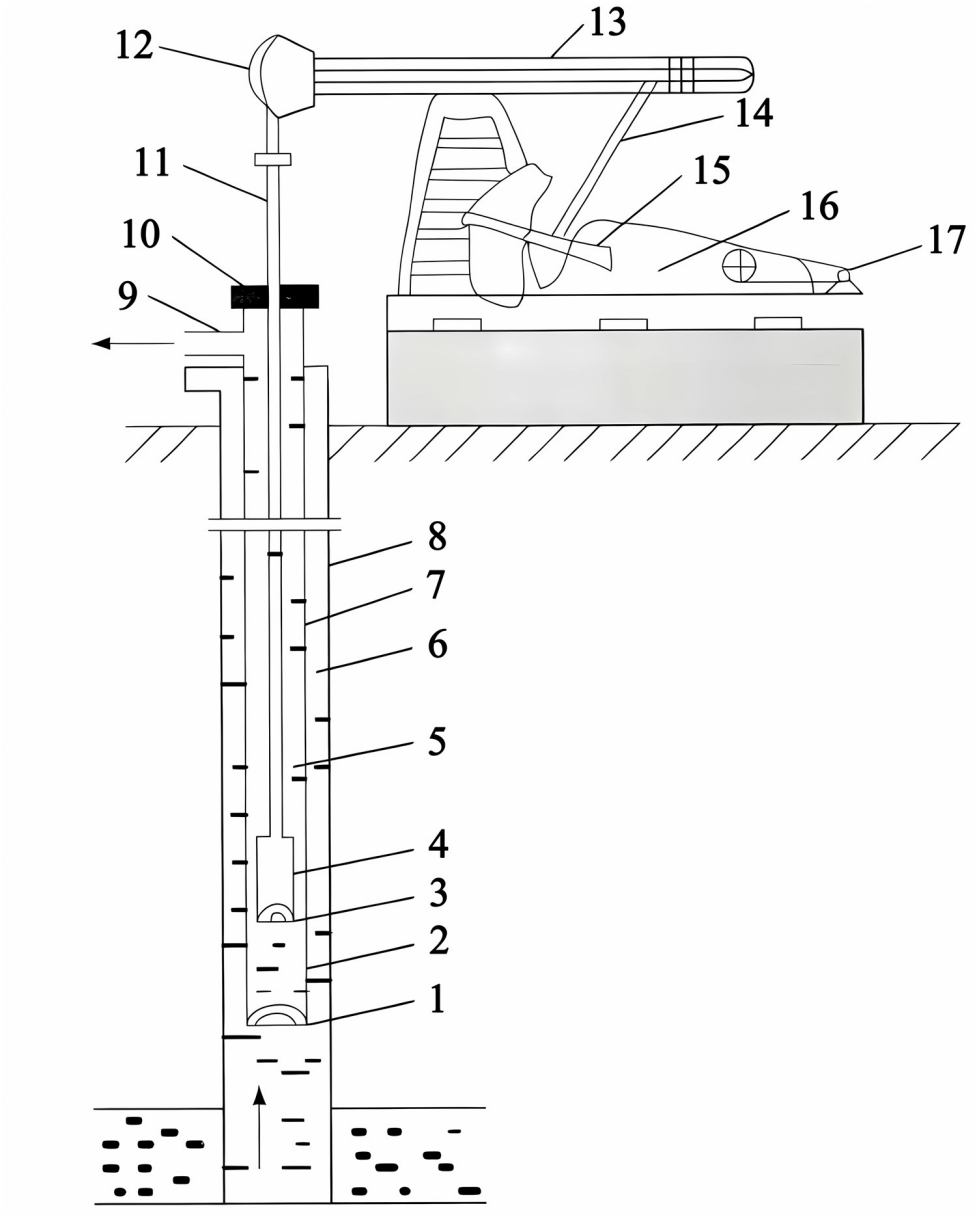
Structure diagram of beam oil pumping unit. Pour: Names of the parts in the diagram: 1. fixed valve; 2. pump barrel; 3. plunger; 4. swing valve; 5. pumping rod; 6. dynamic fluid level; 7. oil pipe; 8. casing; 9. tee; 10. packing box; 11. light rod; 12. donkey head; 13. swing beam; 14. connecting rod; 15. crank; 16. reduction box; 17. electric motor.

The oil pumping machine is a necessary machine in oilfield oil recovery work, which has the advantages of high applicability, reliable operation and low environmental requirements. The structure of oil pumping machine is shown in Fig 6, which mainly consists of four parts: oil beam, support, reducer and distribution box. For example, the oil beam contains the donkey head, oil beam, cross beam, etc. The bracket contains the working ladder, guard ring, etc., the reducer contains the bottom boat, cylinder seat, reducer, etc. And the distribution part contains the motor seat, motor, distribution box, etc. The work diagram shows that in the process of pumping, the light pole is called the upper dead point at the highest point and the lower dead point at the lowest point. Usually, the light rod of the pumping machine is the starting position from the lower dead point, the displacement S is the horizontal axis, and the load F is the vertical axis, and the closed curve about S and F in a pumping process is the work graph of the pumping machine in this pumping cycle.

In the actual work of oil recovery, failures usually occur due to various unforeseen factors such as improper operation, complex environment, accumulation of internal mechanical wear and tear, and corrosion of chemical components. As the pumping unit works downhole, different faults are reflected in the corresponding shape of the power diagram, with which conventional pumping unit fault analysis can be performed. At present, the overall pumping unit failure is categorized into more than 20 types, such as gas impact, sand out of oil well, and leakage of swimming vanes [30–32], and different failures correspond to different shapes of schematic diagrams. In this experiment, 1278 sets of indicator diagram data of 10 common types of pumping units in Shengli Oilfield were selected as experimental samples. The data sample is collected by Shengli Oilfield in the actual production environment for many years, covering the fault types of pumping units in major domestic oilfields, and finally integrated and classified. Because in the domestic oil field, the probability of failure of some pumping units is very small, so combined with the actual production needs of the oil field, this paper mainly studies the common fault diagnosis of pumping units for many years.

During the operation of the pumping unit, such as the failure of the sucker rod, it is mainly due to the elastic fatigue of the sucker rod, and the pump makes the sucker rod string break beyond the tensile yield limit, or the tripping occurs due to the lack of tightening between the sucker rods, which leads to the horizontal strip of the indicator diagram and the oil well does not produce oil.

When the upper stroke begins, the fixed valve opens, and the oil-gas mixture enters the pump. With the upward movement of the piston, the pressure in the pump barrel decreases, and a large amount of gas dissolved in the crude oil is separated. At the same time, due to the expansion of the gas, the polished rod load cannot be quickly increased to the maximum theoretical value, so the loading becomes slower and the slope of the loading line becomes smaller. In the down stroke, due to the gas compression in the pump barrel, the pressure in the pump barrel rises slowly, and the opening time of the traveling valve lags behind, resulting in the slow unloading of the polished rod, and the unloading line is an arc. The greater the curvature of the arc, the lower the pump efficiency. If the oil is thick, when the viscosity of crude oil increases, the additional resistance of the sucker rod becomes larger, which increases the friction between the upper and lower rows of the sucker rod, the upper stroke increases the load, the lower stroke decreases the load, the indicator diagram becomes longer, and the four corners are smooth. The pattern varies, but the working area and filling coefficient of the pump are high, and the drainage is still normal.

If the liquid supply is insufficient, the discharge capacity of the pump is large, the liquid supply capacity of the oil layer is large, the submergence degree is too small, the liquid can not fill the pump barrel, and it can not be unloaded immediately during the down stroke. When the piston contacts the oil level, it is unloaded quickly.

Such as swimming valve leakage, due to the swimming valve assembly is not strict or wear will cause the swimming valve leakage. During the upper stroke, the pressure in the pump is reduced, and the pressure difference between the two ends of the plunger is generated. So that the liquid above the plunger leaks into the working cylinder below the plunger, and the leakage speed increases with the decrease of the pressure below the plunger. Due to the upward ‘jacking’ effect of the liquid leaking below the plunger, the suspension point load cannot rise to the maximum value in time, which slows down the load. With the acceleration of the suspension point movement, the ‘jacking’ effect is relatively reduced until the moment when the upward speed of the plunger is greater than the leakage speed, and the suspension point load reaches the maximum static load. When the plunger continues to rise to the second half stroke, the upward speed of the piston gradually slows down. At the moment when the plunger speed is small and the leakage speed is small, the ‘jacking’ effect of the leakage liquid appears again, which causes the suspension point load to be unloaded in advance.

**Fig 7:**
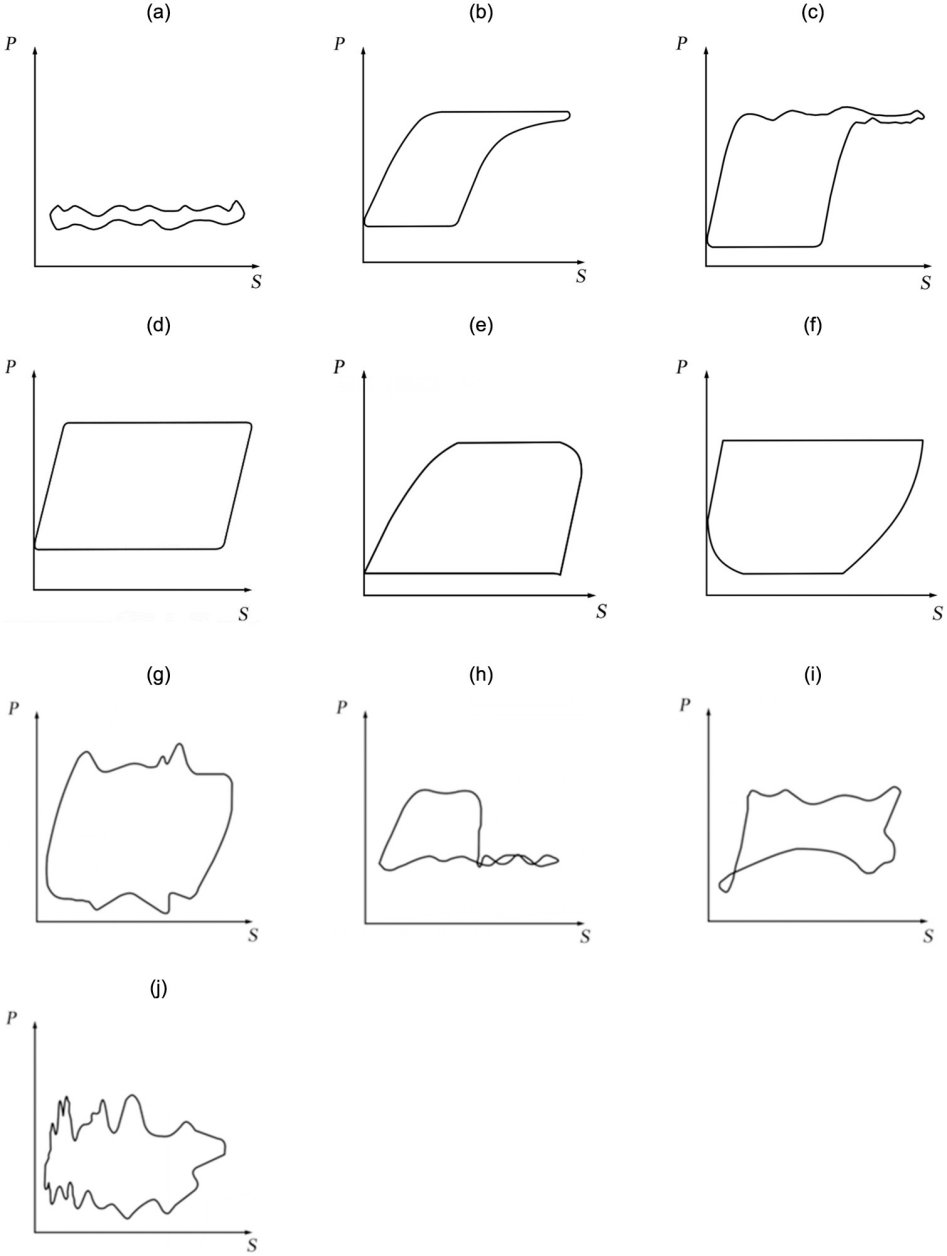
Indicator diagram of pumping unit.

When the stroke of the polished rod of the pumping unit is too large, the plunger is detached during the upper stroke, which will cause a sharp increase in liquid leakage. In Fig 7, the various faults of the pumping unit and the indicator diagram when the pumping unit is in normal working state are shown respectively.

### 5.2 Troubleshooting process

In this experiment, a total of 1278 sets of 10 common types of pumping machine power diagram data from Shengli Oilfield are selected as the experimental samples, and the data are preprocessed using the improved Fourier descriptors of this study. And then combined with the RDC-RBF algorithm to be applied to pumping machine fault diagnosis. Two-thirds of the experimental data are used as the training sample set to train the neural network, and the remaining one-third of the data are used to test the final effect of the proposed method, and the main steps are shown below.

1. First of all, the coordinate points of the contour edge of the schematic figure are extracted and differenced, and then the coordinates of the contour of the schematic figure with equal spacing are obtained.
2. The centre of gravity of the graph is then calculated and the furthest point of the intersection of the minimum inertia axis over the centre of gravity and the contour of the graph is then determined as the starting point.
3. The number of hidden layer neurons of the RBF is determined by finding the point with the highest density and using it as the hidden layer neuron, while introducing the properties of the Gaussian function to ensure the activity of the hidden layer neuron.
4. The parameters of the centre vector, width and connection weights of the RDC-RBF are obtained by using CS with the introduction of dynamic discovery probability and adaptive step improvement.
5. The number of neurons in the output layer is specified to be 10 because the sample data set of this experiment includes the normal state of the pumping machine and nine common fault types.
6. The final DACS-RDC-RBF model was used to diagnose the fault of the pumping unit after training, and the closest one was the fault type of the pumping unit according to the diagnostic results of the output layer compared with the pumping unit fault numbers recorded in Table 1.

**Table 1:** Pumping machine working condition type number table.

Condition number	Fault Type	0	1	2	3	4	5	6	7	8	9
Type Zero	Normal	O	×	×	×	×	×	×	×	×	×
Type One	Atmospheric influences	×	O	×	×	×	×	×	×	×	×
Type Two	Insufficient fluid supply	×	×	O	×	×	×	×	×	×	×
Type Three	Oil well out of sand	×	×	×	O	×	×	×	×	×	×
Type Four	Fixed Vanier Leakage	×	×	×	×	O	×	×	×	×	×
Type Five	Touring vanilla leakage loss	×	×	×	×	×	O	×	×	×	×
Type Six	Oil well waxing	×	×	×	×	×	×	O	×	×	×
Type Seven	Pumping rod disconnected	×	×	×	×	×	×	×	O	×	×
Type Eight	Bump pumps	×	×	×	×	×	×	×	×	O	×
Type Nine	Plunger dislodged	×	×	×	×	×	×	×	×	×	O

**Fig 8:**
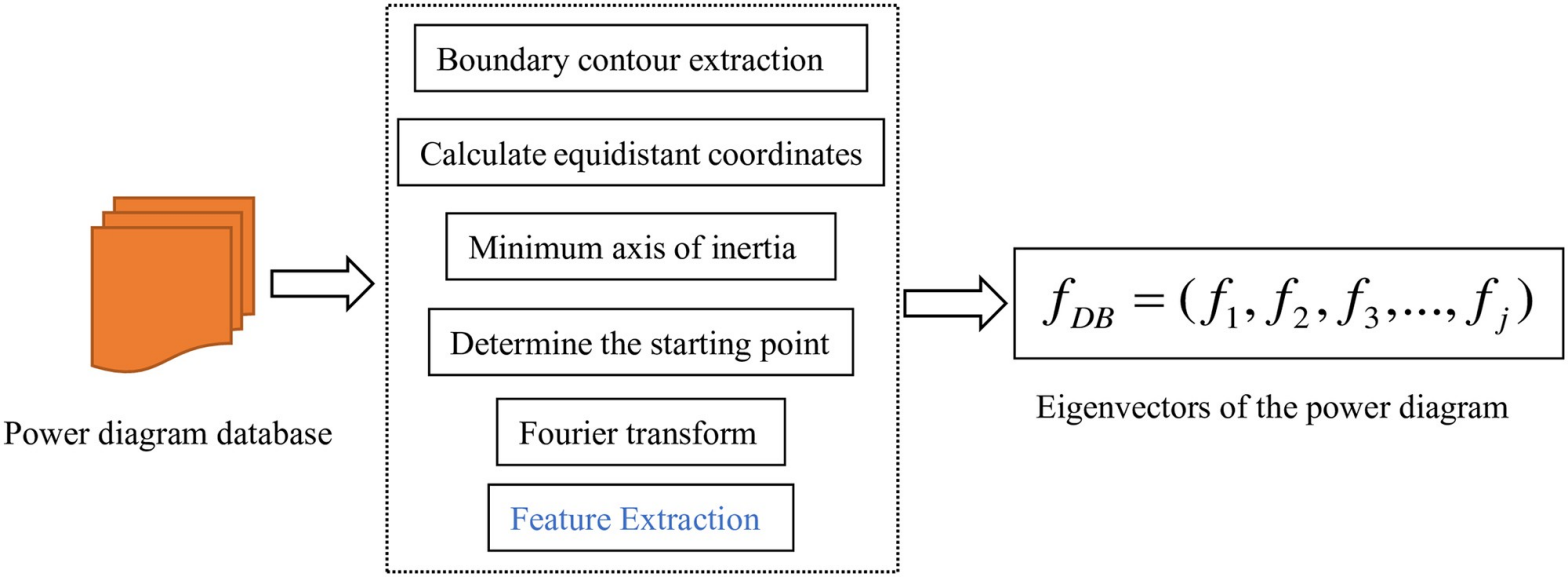
The process of indicator diagram feature extraction.

**Fig 9:**
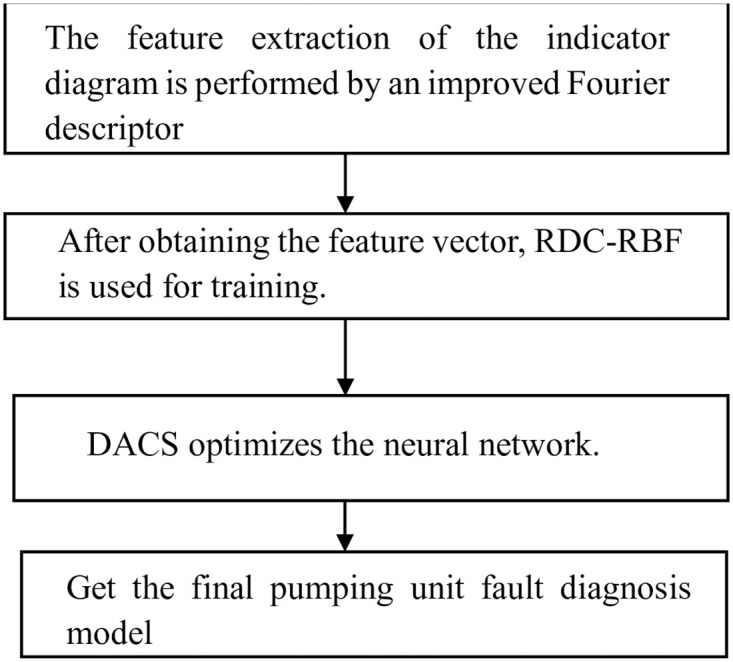
Experimental process diagram.

In the above steps, for the pumping machine power diagram, the feature extraction process using the improved Fourier descriptor is shown in Fig 8. And the final extracted pumping machine power diagram feature vectors are shown in Table 2 below, which are used as the input of the RDC-RBF neural network. Firstly, the improved Fourier descriptor is used to extract the feature of the indicator diagram data, and then the obtained feature vector is used as the input layer of the improved RDC-RBF neural network. The final fault category is used as the output to train the neural network. In this process, the improved DACS is used to optimize the parameters of the neural network. Finally, the fault diagnosis model of pumping unit is obtained. The experimental process is shown in Fig 9.

**Table 2:** Characteristic vector of indicator diagram.

Fault Type	*f*_1_	*f*_2_	*f*_3_	*f*_4_	*f*_5_	*f*_6_	*f*_7_
Normal	0.5308	0.3736	0.4328	0.8793	0.4231	0.3805	0.5472
Atmospheric influences	0.3628	0.4674	0.5137	0.8623	0.5203	0.4612	0.3722
Insufficient fluid supply	0.4611	0.4623	0.4121	0.8987	0.4174	0.4629	0.4512
Oil well out of sand	0.3510	0.4398	0.5102	0.9003	0.4998	0.4415	0.3517
Fixed Vanier Leakage	0.3397	0.4128	0.5309	0.8837	0.5308	0.4128	0.3407
Touring vanilla leakage loss	0.3928	0.5344	0.4112	0.8839	0.4104	0.5366	0.3926
Oil well waxing	0.3483	0.4428	0.5107	0.8945	0.5128	0.4329	0.3482
Pumping rod disconnected	0.4010	0.5197	0.4042	0.9018	0.4128	0.5272	0.4103
Bump pumps	0.3529	0.4541	0.5253	0.8986	0.5249	0.4548	0.3537
Plunger dislodged	0.3958	0.5701	0.4016	0.8907	0.4002	0.5186	0.3989

### 5.3 Algorithm evaluation indicators

For the accuracy of the analysis algorithm, the accuracy rate, the completion rate, and the ROC curve (Receiver Operating Characteristic Curve) [33] are often used in classification models, so they are chosen as evaluation indicators for the final effect of this pumping machine fault diagnosis. And the superiority of the proposed diagnostic model is verified based on the evaluation results. According to the confusion matrix formed by the evaluation indexes, as shown in Table 3, all data samples are divided into a total of four cases according to their true and predicted categories: true cases (*TP*), false positive cases (*FP*), true negative cases (*TN*), and false negative cases (*FN*) [34], and in the pumping machine fault diagnosis problem, in each fault category, the corresponding ROC curves can be obtained, which are finally obtained by The final ROC curve is obtained by “micro-average” for the evaluation of the model.

**Table 3:** Confusion matrix of classification results.

The Real Deal	Predicted results	
	Positive	Negative
Positive (True)	*TP*	*TN*
Negative(False)	*FP*	*FN*

In the “micro-averaging method”, we will calculate performance from individual true positives, true negatives, false positives and false negatives for multiple categories of pumping unit fault diagnosis models, e.g. accuracy:

$$\begin{array}{c} PRE_{\text{micro}}=\frac{TP_{1}+\cdots +TP_{k}}{TP_{1}+\cdots +TP_{k}+FP_{1}+\cdots +FP_{k}} \end{array}$$ (33)

### 5.4 Experimental results

In order to verify the actual detection effect and superiority of the improved Fourier descriptor combined with RDC-RBF pumping unit fault diagnosis method proposed in the study, this paper uses 1278 sets of 10 common types of pumping unit dynamometer data in Shengli Oilfield. This set of data is collected by Shengli Oilfield in the production process of oil fields in recent years, and is composed of screening, classification and data enhancement. In this study, DACS-RDC-RBF and DACS-RDC-RBF, PSO-BP, UKF-RBF, CS-BP, CNN and other mainstream methods currently used in fault diagnosis of pumping units are used respectively 90% of the data are randomly used as training sets for model training and testing, and 10% of the data are selected as validation sets to verify the final results. By testing on the same platform, and then a comprehensive comparison. This experiment is based on the NVIDIA RTX 3080 GPU hardware environment, using TensorFlow, sklearn, tflearn as a development platform, the system is Windows 10.

**Fig 10:**
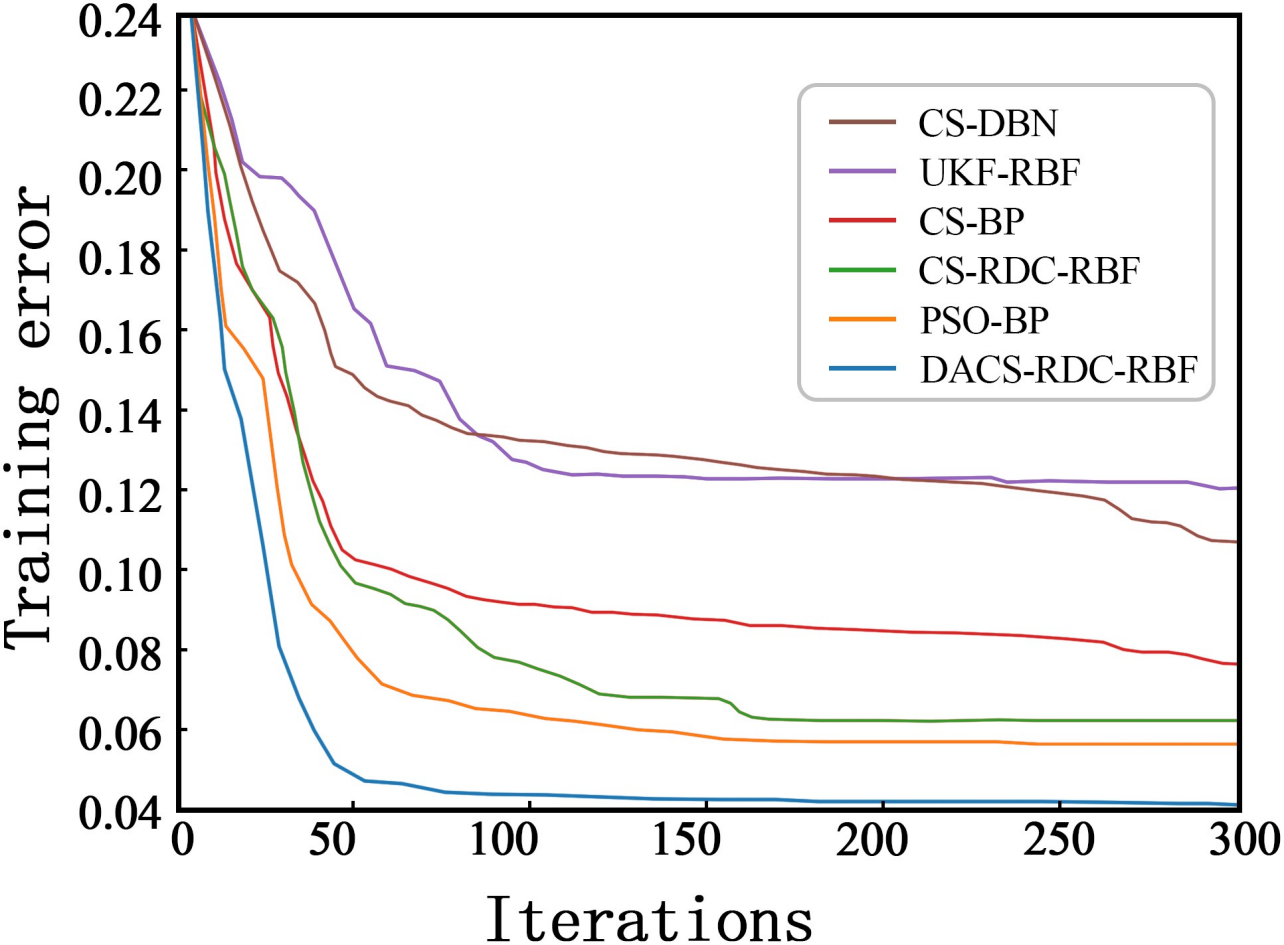
Comparison of algorithm search curves.

After the experiments, the training error and the number of iterations of the algorithms are compared as shown in Fig 10. In the same case, by comparing RDC-RBF with DACS-RDC-RBF, it is shown that the CS algorithm after the research improvement has gained significant improvement in the search capability. When compared as a whole, DACS-RDC-RBF has a better speed in the early stage of the search, while gradually leveling off after about 50 iterations and obtaining the global optimum, proving that DACS has the fastest and best search speed.

**Fig 11:**
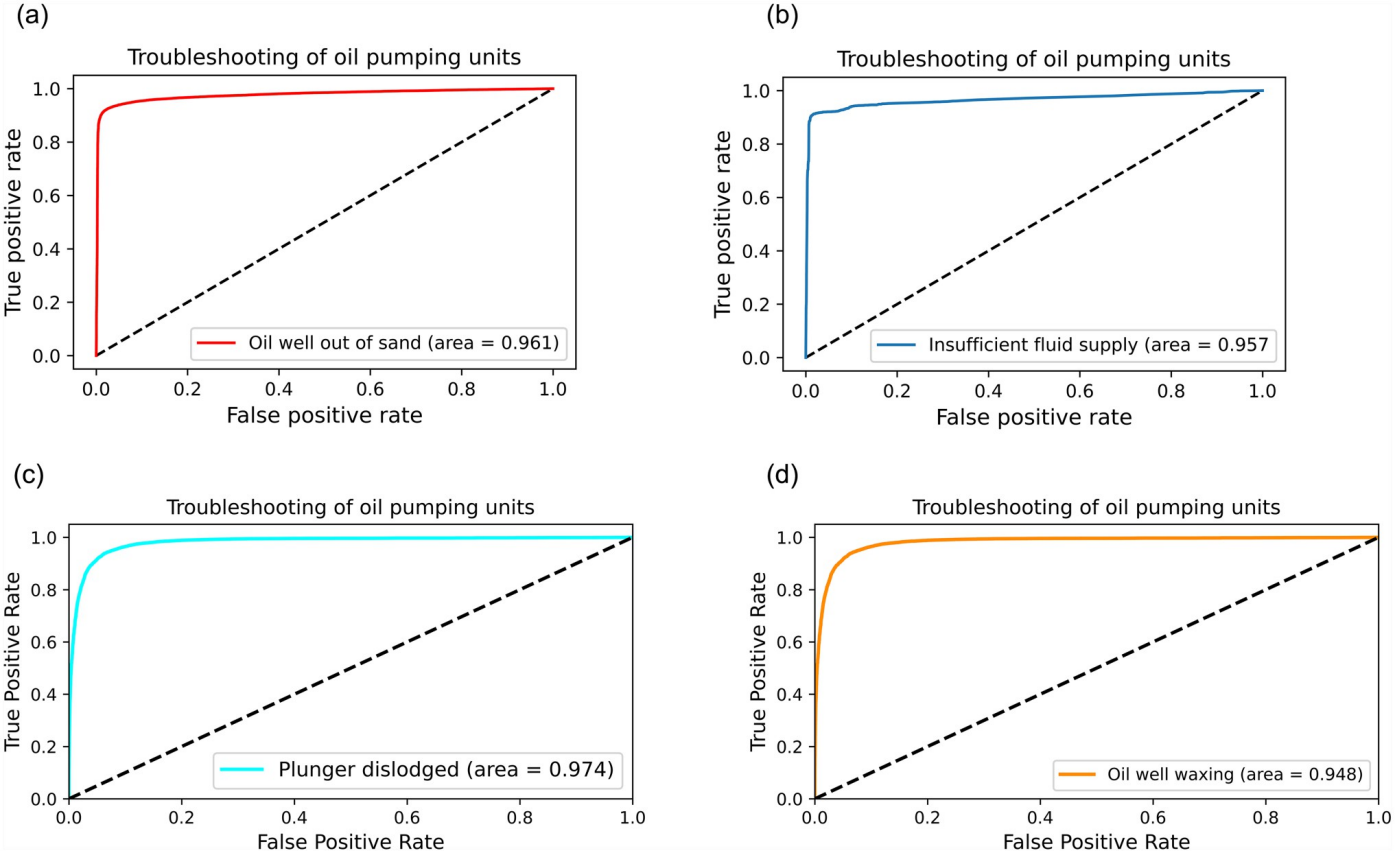
ROC evaluation chart of some categories. The black dotted line is the reference line, and the dotted line reflects the result of random selection.

**Fig 12:**
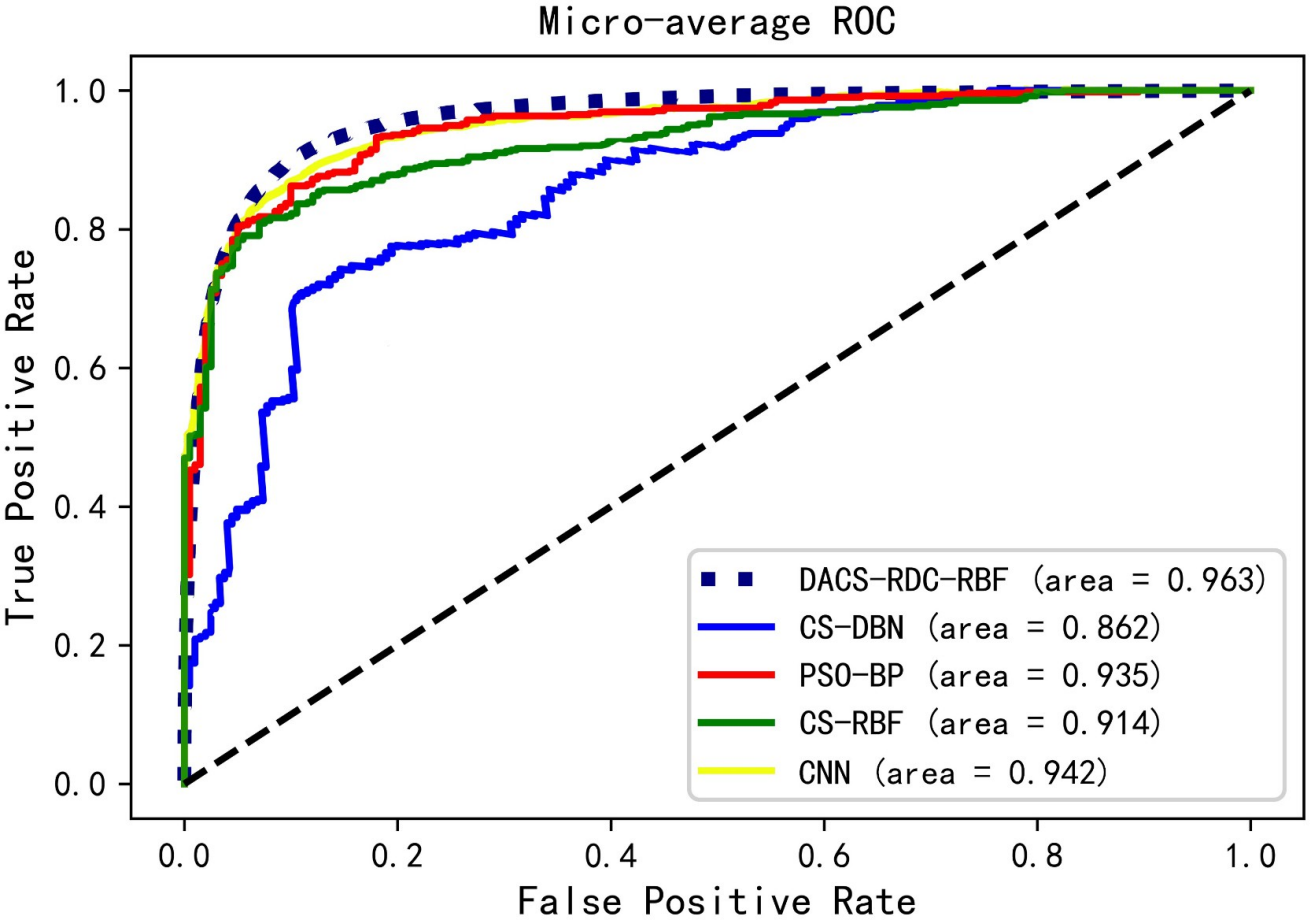
micro-average ROC of model. The black dotted line is the reference line, and the dotted line reflects the result of random selection.

The area under the ROC curve AUC (Area Under ROC Curve) is used as a standard to evaluate the final diagnostic effect of the DACS-RDC-RBF model. The diagnostic effect of the algorithm is proportional to the AUC area. The larger the AUC area, the better the diagnostic effect of the algorithm. When comparing different fault diagnosis models, the ROC curve is intuitive to evaluate the test effect of the algorithm. When the ROC curve area of one classifier can completely include the curve area of the other classifier, it can be concluded that the diagnosis effect of the former is better than the latter. Figs 11 and 12 show the fault diagnosis model of pumping unit DACS-RDC-RBF in Insufficient fluid supply, Oil well out of sand, Oil well waxing, Plunger dislodged, Atmospheric influences, Touring vanilla leakage loss, Fixed Vanier Leakage and other categories. And the final microscopic average ROC curve results were evaluated and compared. From the diagram, it can be seen that after improving the RBF structure based on the fastest density clustering, excellent results have been achieved for the diagnosis of pumping unit faults. The availability of the proposed method is demonstrated by ROC curve analysis.

**Fig 13:**
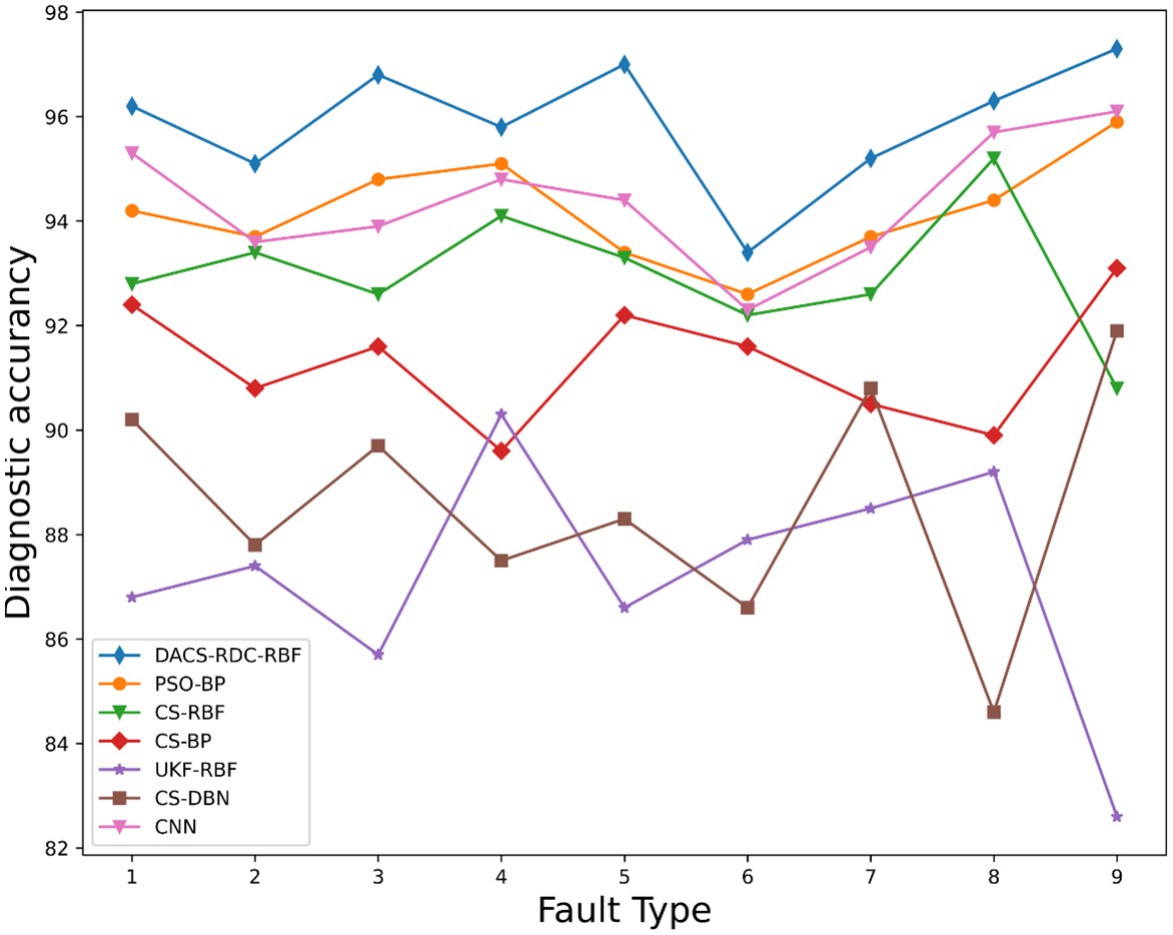
Algorithm diagnostic accuracy comparison chart. Names of the parts in the diagram: 1. Atmospheric influences; 2. Insufficient fluid supply; 3. Oil well out of sand; 4. Fixed Vanier Leakage; 5. Touring vanilla leakage loss; 6. Oil well waxing; 7. Pumping rod disconnected; 8. Bump pumps; 9. Plunger dislodged;.

Fig 13 shows the comparison of the accuracy curves of the nine fault types of the pumping unit selected in this experiment after the data processing method of the improved Fourier descriptor is adopted for the six diagnostic models. The coordinates of the horizontal axis correspond to the pumping unit fault type number in Table 1. According to the test results, it can be seen that the overall diagnostic level of DACS-RDC-RBF is the highest, and the diagnostic accuracy for various fault types can meet the current practical application requirements. Combined with the average diagnostic accuracy and average training time of each model shown in Table 4 and Fig 13. These show that the improved DACS-RDC-RBF pumping unit fault diagnosis method reduces the overall time consumption of the model. Under the same hardware resources and experimental platform, it is verified that the proposed method can diagnose the pumping unit fault more efficiently. Although the accuracy of CNN algorithm can meet the requirements, its training time is too long. And in practical application, the working condition detection equipment of pumping unit can not run well. And the average accuracy of DACS-RDC-RBF detection can reach about 96.3%, which reflects that the optimized RBF is more suitable for fault diagnosis of pumping units than DBN and BP, and the improved Fourier descriptor is used to process the dynamometer diagram of pumping units. The diagnostic accuracy of the experiment has been improved.

**Table 4:** Pumping machine fault diagnosis results table.

Diagnostic model	Average detection accuracy	Average training time
(Improved Fourier descriptors)111DACS-RDC-RBF	96.3%	8min
(Improved Fourier descriptors)111PSO-BP	94.8%	10min
(Improved Fourier descriptors)111CS-RBF	93.4%	14min
(Improved Fourier descriptors)111CS-BP	92.1%	13min
(Improved Fourier descriptors)111UKF-RBF	88.3%	13min
(Improved Fourier descriptors)111CS-DBN	88.7%	14min
(Fourier descriptors)111DACS-RDC-RBF	92.4%	9min
(Fourier descriptors)111CS-RBF	89.7%	14min
(Improved Fourier descriptors)111CNN	94.4%	28.7h

## 6 Conclusion

In this paper, a new diagnosis method based on improved Fourier descriptor and RDC-RBF is proposed for the current fault diagnosis problem of pumping unit. At the same time, the improved dynamic adaptive cuckoo search is used to optimize the RDC-RBF model. After the experiment of the pumping unit indicator diagram data set, the following conclusions can be drawn:

1. The starting point position of the schematic contours is first determined by the minimum inertia axis to achieve the best matching between contours. The resulting feature vectors can be extracted to better characterise the schematic features than the original Fourier descriptors.
2. By ensuring neuron activity and seeking the highest density of points as hidden layer neurons, the resulting RBF neural network structure is more effective than the original RBF in diagnosing pumping machine faults.
3. By introducing dynamic discovery probability and adaptive step improvement of cuckoo search, the optimized neural network accelerates the convergence speed and obtains the optimal parameters of RBF faster.

Finally, the RDC-RBF diagnostic model is used for fault diagnosis of pumping units and compared with mainstream diagnostic methods (PSO-BP, UKF-RBF, CS-BP,CNN, etc.). And the superiority of this method is demonstrated, and the final average accuracy is about 96.3%. Although the research can satisfy the production work of some domestic oil fields in China, the method still has some limitations for some severe conditions and the occurrence of fault types not mentioned in the paper. Therefore, in the future, further experimental research can be carried out with more comprehensive information, and breakthroughs can also be sought in emerging areas such as artificial ant colony optimization algorithms, mechanical fault diagnosis with digital twins, and review of degradation models.

## Supporting information

S1 Data DiagramDataLiBowen. (ZIP)
