# Supplementary figures and images for: Pumping machine fault diagnosis based on fused RDC-RBF (part 2 of 2)
Source: PLoS One. 2023 Sep 25;18(9):e0291777. doi: 10.1371/journal.pone.0291777 (PMC10519606; doi:10.1371/journal.pone.0291777)

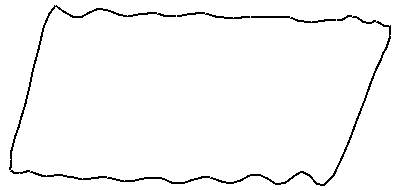

Supplement: S1 Data — (ZIP) [file pone.0291777.s001.zip › DiagramDataLiBowen/A01/A0101_73.png]

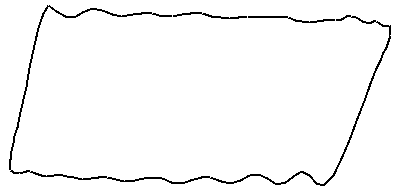

Supplement: S1 Data — (ZIP) [file pone.0291777.s001.zip › DiagramDataLiBowen/A01/A0101_74.png]

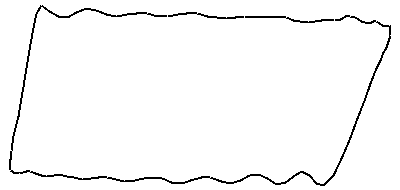

Supplement: S1 Data — (ZIP) [file pone.0291777.s001.zip › DiagramDataLiBowen/A01/A0101_75.png]

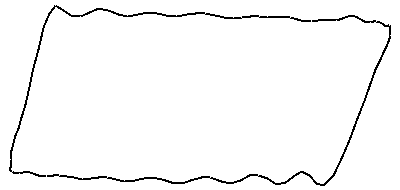

Supplement: S1 Data — (ZIP) [file pone.0291777.s001.zip › DiagramDataLiBowen/A01/A0101_76.png]

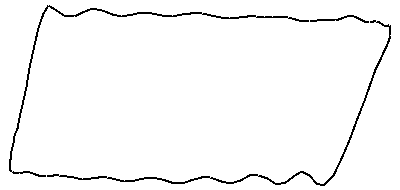

Supplement: S1 Data — (ZIP) [file pone.0291777.s001.zip › DiagramDataLiBowen/A01/A0101_77.png]

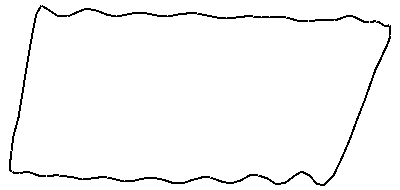

Supplement: S1 Data — (ZIP) [file pone.0291777.s001.zip › DiagramDataLiBowen/A01/A0101_78.png]

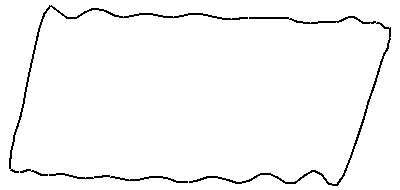

Supplement: S1 Data — (ZIP) [file pone.0291777.s001.zip › DiagramDataLiBowen/A01/A0101_8.png]

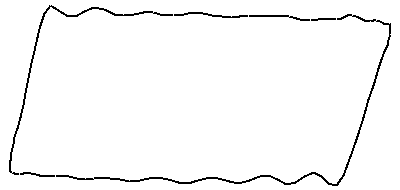

Supplement: S1 Data — (ZIP) [file pone.0291777.s001.zip › DiagramDataLiBowen/A01/A0101_83.png]

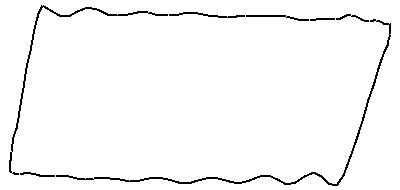

Supplement: S1 Data — (ZIP) [file pone.0291777.s001.zip › DiagramDataLiBowen/A01/A0101_84.png]

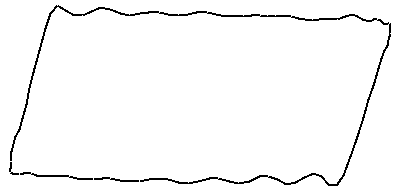

Supplement: S1 Data — (ZIP) [file pone.0291777.s001.zip › DiagramDataLiBowen/A01/A0101_85.png]

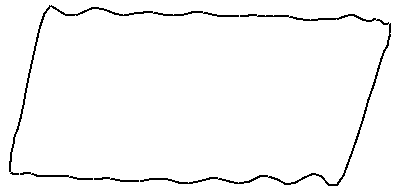

Supplement: S1 Data — (ZIP) [file pone.0291777.s001.zip › DiagramDataLiBowen/A01/A0101_86.png]

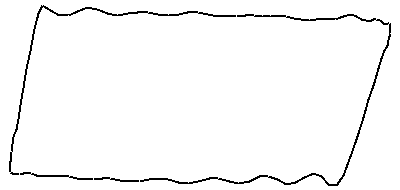

Supplement: S1 Data — (ZIP) [file pone.0291777.s001.zip › DiagramDataLiBowen/A01/A0101_87.png]

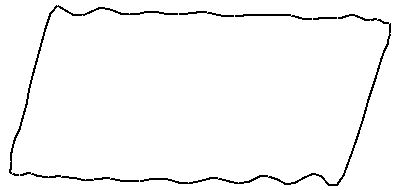

Supplement: S1 Data — (ZIP) [file pone.0291777.s001.zip › DiagramDataLiBowen/A01/A0101_88.png]

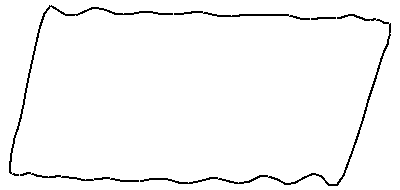

Supplement: S1 Data — (ZIP) [file pone.0291777.s001.zip › DiagramDataLiBowen/A01/A0101_89.png]

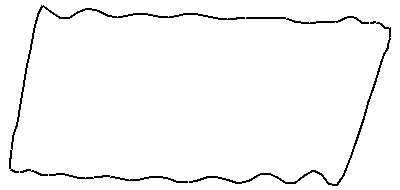

Supplement: S1 Data — (ZIP) [file pone.0291777.s001.zip › DiagramDataLiBowen/A01/A0101_9.png]

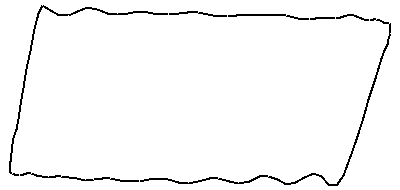

Supplement: S1 Data — (ZIP) [file pone.0291777.s001.zip › DiagramDataLiBowen/A01/A0101_90.png]

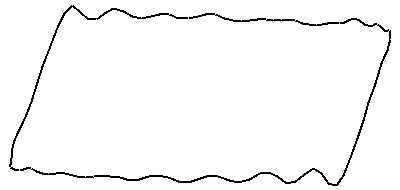

Supplement: S1 Data — (ZIP) [file pone.0291777.s001.zip › DiagramDataLiBowen/A01/A0101_91.png]

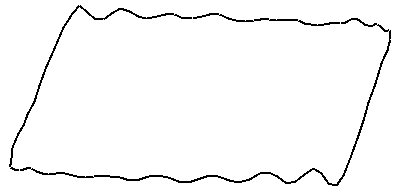

Supplement: S1 Data — (ZIP) [file pone.0291777.s001.zip › DiagramDataLiBowen/A01/A0101_92.png]

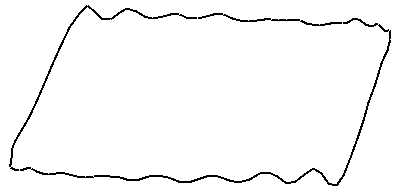

Supplement: S1 Data — (ZIP) [file pone.0291777.s001.zip › DiagramDataLiBowen/A01/A0101_93.png]

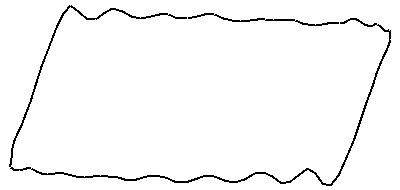

Supplement: S1 Data — (ZIP) [file pone.0291777.s001.zip › DiagramDataLiBowen/A01/A0101_94.png]

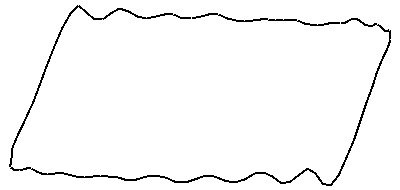

Supplement: S1 Data — (ZIP) [file pone.0291777.s001.zip › DiagramDataLiBowen/A01/A0101_95.png]

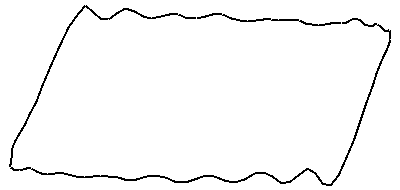

Supplement: S1 Data — (ZIP) [file pone.0291777.s001.zip › DiagramDataLiBowen/A01/A0101_96.png]

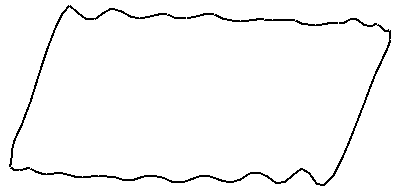

Supplement: S1 Data — (ZIP) [file pone.0291777.s001.zip › DiagramDataLiBowen/A01/A0101_97.png]

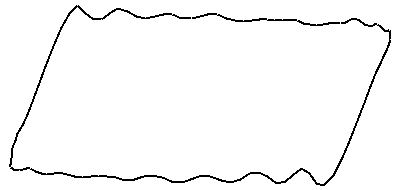

Supplement: S1 Data — (ZIP) [file pone.0291777.s001.zip › DiagramDataLiBowen/A01/A0101_98.png]

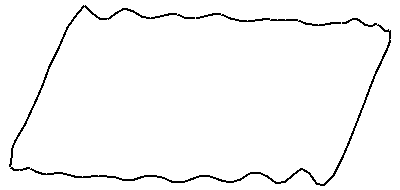

Supplement: S1 Data — (ZIP) [file pone.0291777.s001.zip › DiagramDataLiBowen/A01/A0101_99.png]

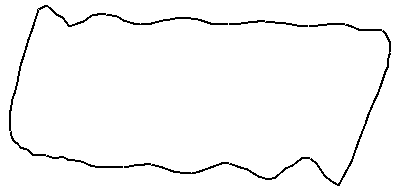

Supplement: S1 Data — (ZIP) [file pone.0291777.s001.zip › DiagramDataLiBowen/A01/A0102_1.png]

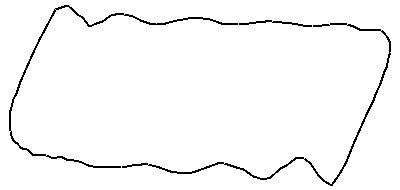

Supplement: S1 Data — (ZIP) [file pone.0291777.s001.zip › DiagramDataLiBowen/A01/A0102_10.png]

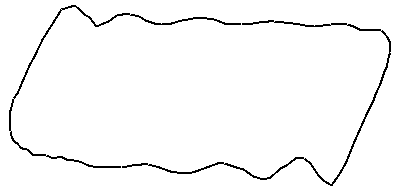

Supplement: S1 Data — (ZIP) [file pone.0291777.s001.zip › DiagramDataLiBowen/A01/A0102_11.png]

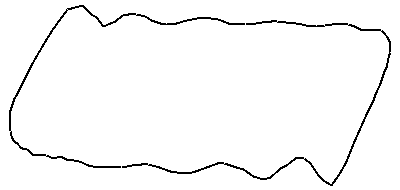

Supplement: S1 Data — (ZIP) [file pone.0291777.s001.zip › DiagramDataLiBowen/A01/A0102_12.png]

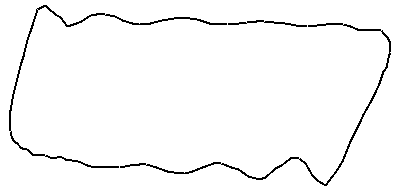

Supplement: S1 Data — (ZIP) [file pone.0291777.s001.zip › DiagramDataLiBowen/A01/A0102_13.png]

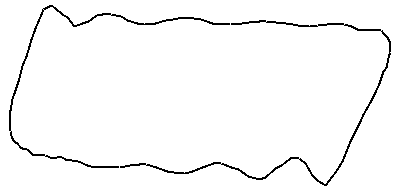

Supplement: S1 Data — (ZIP) [file pone.0291777.s001.zip › DiagramDataLiBowen/A01/A0102_14.png]

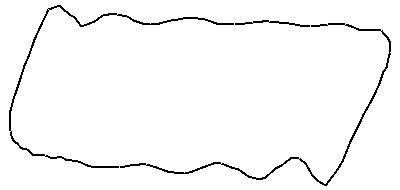

Supplement: S1 Data — (ZIP) [file pone.0291777.s001.zip › DiagramDataLiBowen/A01/A0102_15.png]

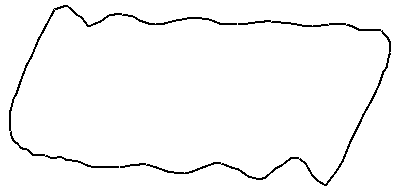

Supplement: S1 Data — (ZIP) [file pone.0291777.s001.zip › DiagramDataLiBowen/A01/A0102_16.png]

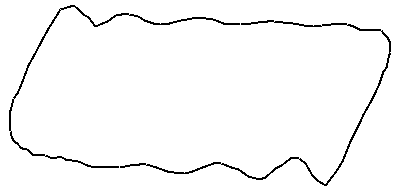

Supplement: S1 Data — (ZIP) [file pone.0291777.s001.zip › DiagramDataLiBowen/A01/A0102_17.png]

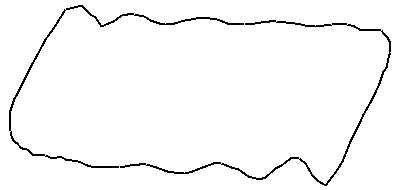

Supplement: S1 Data — (ZIP) [file pone.0291777.s001.zip › DiagramDataLiBowen/A01/A0102_18.png]

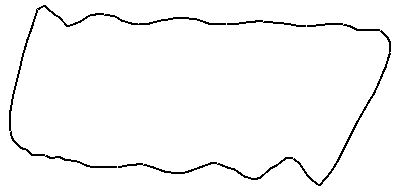

Supplement: S1 Data — (ZIP) [file pone.0291777.s001.zip › DiagramDataLiBowen/A01/A0102_19.png]

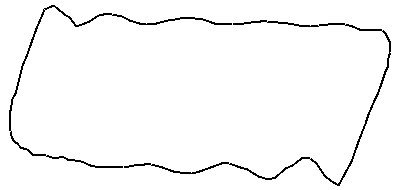

Supplement: S1 Data — (ZIP) [file pone.0291777.s001.zip › DiagramDataLiBowen/A01/A0102_2.png]

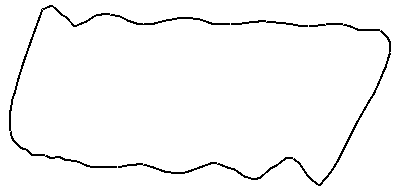

Supplement: S1 Data — (ZIP) [file pone.0291777.s001.zip › DiagramDataLiBowen/A01/A0102_20.png]

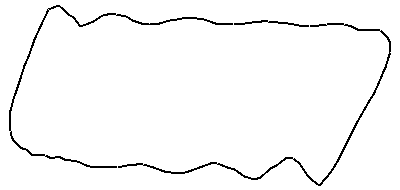

Supplement: S1 Data — (ZIP) [file pone.0291777.s001.zip › DiagramDataLiBowen/A01/A0102_21.png]

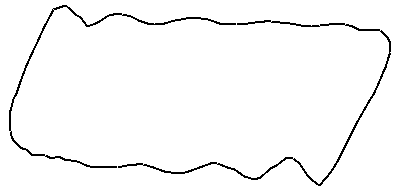

Supplement: S1 Data — (ZIP) [file pone.0291777.s001.zip › DiagramDataLiBowen/A01/A0102_22.png]

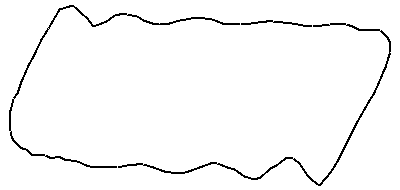

Supplement: S1 Data — (ZIP) [file pone.0291777.s001.zip › DiagramDataLiBowen/A01/A0102_23.png]

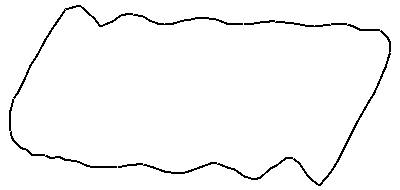

Supplement: S1 Data — (ZIP) [file pone.0291777.s001.zip › DiagramDataLiBowen/A01/A0102_24.png]

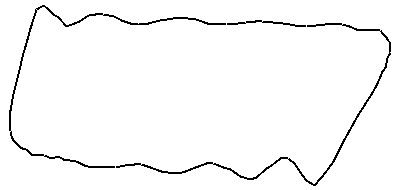

Supplement: S1 Data — (ZIP) [file pone.0291777.s001.zip › DiagramDataLiBowen/A01/A0102_25.png]

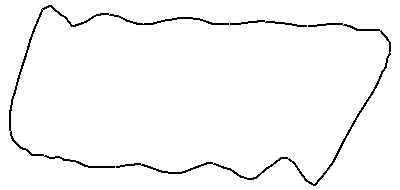

Supplement: S1 Data — (ZIP) [file pone.0291777.s001.zip › DiagramDataLiBowen/A01/A0102_26.png]

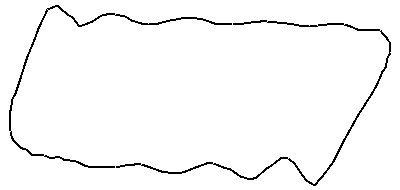

Supplement: S1 Data — (ZIP) [file pone.0291777.s001.zip › DiagramDataLiBowen/A01/A0102_27.png]

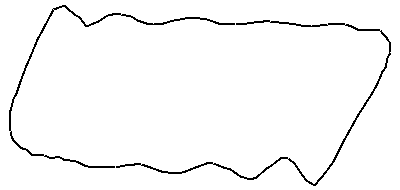

Supplement: S1 Data — (ZIP) [file pone.0291777.s001.zip › DiagramDataLiBowen/A01/A0102_28.png]

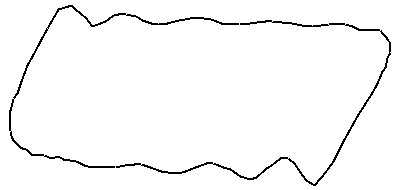

Supplement: S1 Data — (ZIP) [file pone.0291777.s001.zip › DiagramDataLiBowen/A01/A0102_29.png]

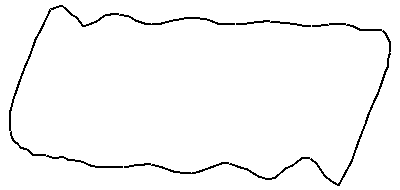

Supplement: S1 Data — (ZIP) [file pone.0291777.s001.zip › DiagramDataLiBowen/A01/A0102_3.png]

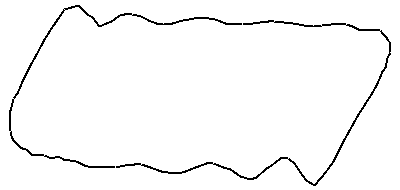

Supplement: S1 Data — (ZIP) [file pone.0291777.s001.zip › DiagramDataLiBowen/A01/A0102_30.png]

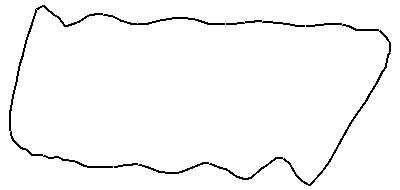

Supplement: S1 Data — (ZIP) [file pone.0291777.s001.zip › DiagramDataLiBowen/A01/A0102_31.png]

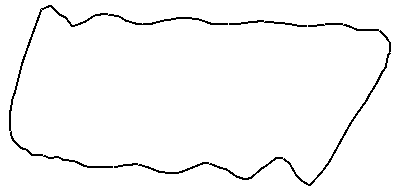

Supplement: S1 Data — (ZIP) [file pone.0291777.s001.zip › DiagramDataLiBowen/A01/A0102_32.png]

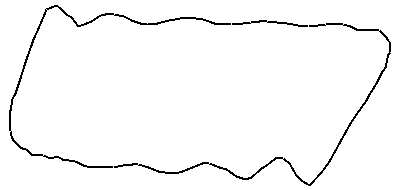

Supplement: S1 Data — (ZIP) [file pone.0291777.s001.zip › DiagramDataLiBowen/A01/A0102_33.png]

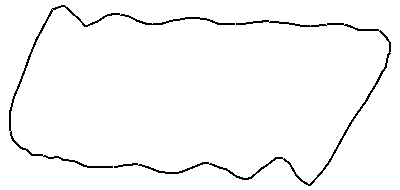

Supplement: S1 Data — (ZIP) [file pone.0291777.s001.zip › DiagramDataLiBowen/A01/A0102_34.png]

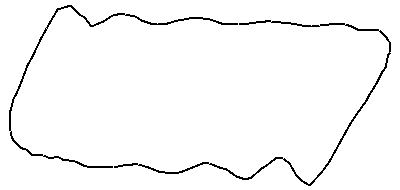

Supplement: S1 Data — (ZIP) [file pone.0291777.s001.zip › DiagramDataLiBowen/A01/A0102_35.png]

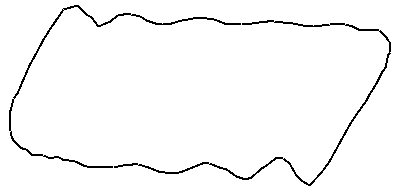

Supplement: S1 Data — (ZIP) [file pone.0291777.s001.zip › DiagramDataLiBowen/A01/A0102_36.png]

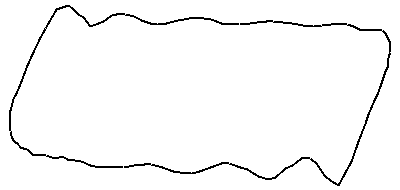

Supplement: S1 Data — (ZIP) [file pone.0291777.s001.zip › DiagramDataLiBowen/A01/A0102_4.png]

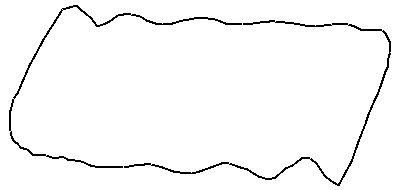

Supplement: S1 Data — (ZIP) [file pone.0291777.s001.zip › DiagramDataLiBowen/A01/A0102_5.png]

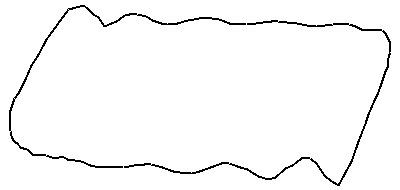

Supplement: S1 Data — (ZIP) [file pone.0291777.s001.zip › DiagramDataLiBowen/A01/A0102_6.png]

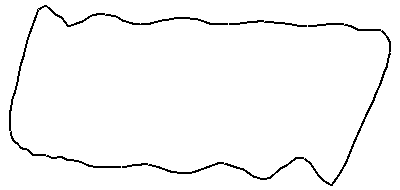

Supplement: S1 Data — (ZIP) [file pone.0291777.s001.zip › DiagramDataLiBowen/A01/A0102_7.png]

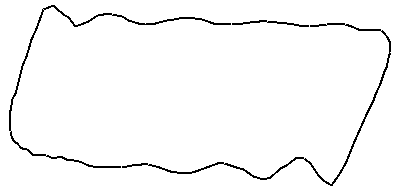

Supplement: S1 Data — (ZIP) [file pone.0291777.s001.zip › DiagramDataLiBowen/A01/A0102_8.png]

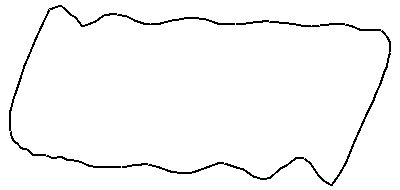

Supplement: S1 Data — (ZIP) [file pone.0291777.s001.zip › DiagramDataLiBowen/A01/A0102_9.png]

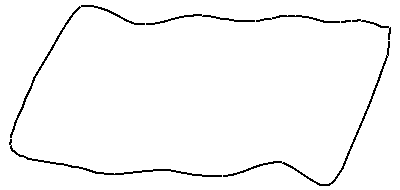

Supplement: S1 Data — (ZIP) [file pone.0291777.s001.zip › DiagramDataLiBowen/A01/A0103_1.png]

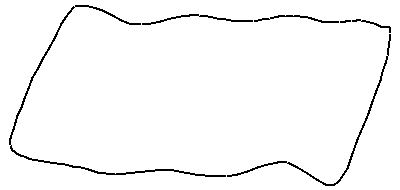

Supplement: S1 Data — (ZIP) [file pone.0291777.s001.zip › DiagramDataLiBowen/A01/A0103_10.png]

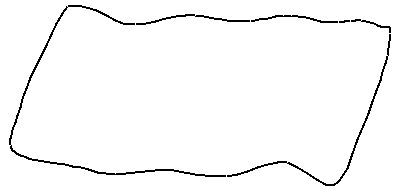

Supplement: S1 Data — (ZIP) [file pone.0291777.s001.zip › DiagramDataLiBowen/A01/A0103_11.png]

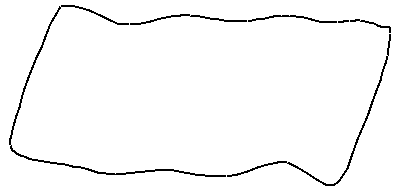

Supplement: S1 Data — (ZIP) [file pone.0291777.s001.zip › DiagramDataLiBowen/A01/A0103_12.png]

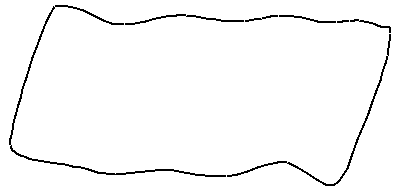

Supplement: S1 Data — (ZIP) [file pone.0291777.s001.zip › DiagramDataLiBowen/A01/A0103_13.png]

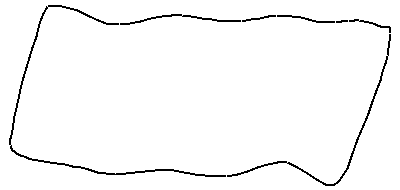

Supplement: S1 Data — (ZIP) [file pone.0291777.s001.zip › DiagramDataLiBowen/A01/A0103_14.png]

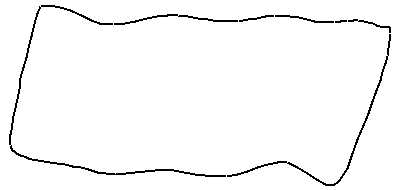

Supplement: S1 Data — (ZIP) [file pone.0291777.s001.zip › DiagramDataLiBowen/A01/A0103_15.png]

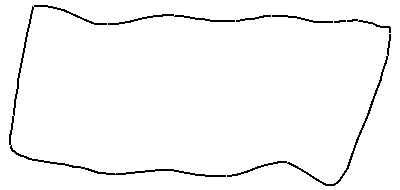

Supplement: S1 Data — (ZIP) [file pone.0291777.s001.zip › DiagramDataLiBowen/A01/A0103_16.png]

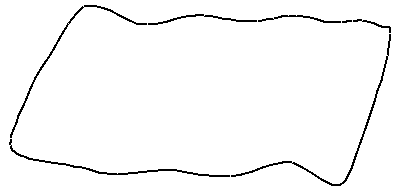

Supplement: S1 Data — (ZIP) [file pone.0291777.s001.zip › DiagramDataLiBowen/A01/A0103_17.png]

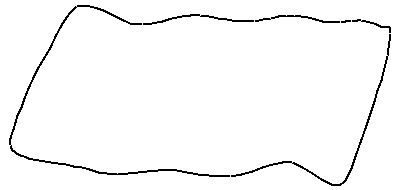

Supplement: S1 Data — (ZIP) [file pone.0291777.s001.zip › DiagramDataLiBowen/A01/A0103_18.png]

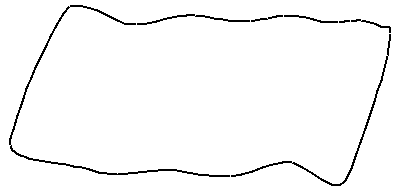

Supplement: S1 Data — (ZIP) [file pone.0291777.s001.zip › DiagramDataLiBowen/A01/A0103_19.png]

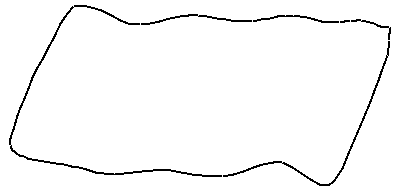

Supplement: S1 Data — (ZIP) [file pone.0291777.s001.zip › DiagramDataLiBowen/A01/A0103_2.png]

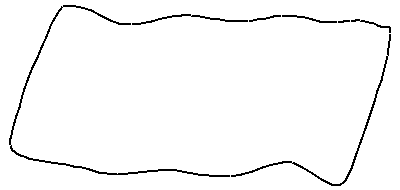

Supplement: S1 Data — (ZIP) [file pone.0291777.s001.zip › DiagramDataLiBowen/A01/A0103_20.png]

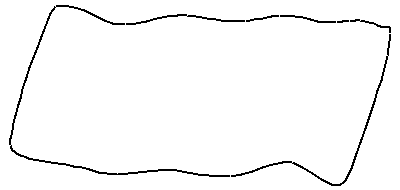

Supplement: S1 Data — (ZIP) [file pone.0291777.s001.zip › DiagramDataLiBowen/A01/A0103_21.png]

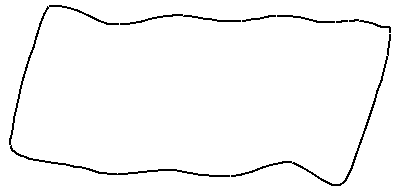

Supplement: S1 Data — (ZIP) [file pone.0291777.s001.zip › DiagramDataLiBowen/A01/A0103_22.png]

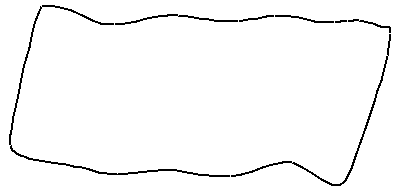

Supplement: S1 Data — (ZIP) [file pone.0291777.s001.zip › DiagramDataLiBowen/A01/A0103_23.png]

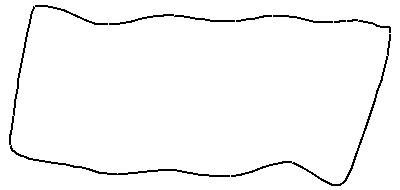

Supplement: S1 Data — (ZIP) [file pone.0291777.s001.zip › DiagramDataLiBowen/A01/A0103_24.png]

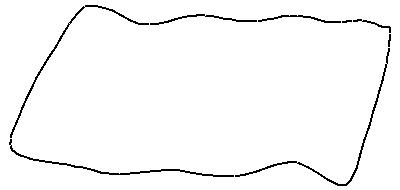

Supplement: S1 Data — (ZIP) [file pone.0291777.s001.zip › DiagramDataLiBowen/A01/A0103_25.png]

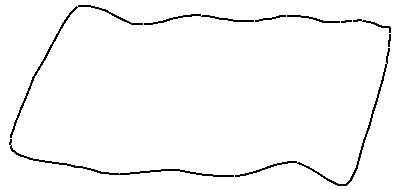

Supplement: S1 Data — (ZIP) [file pone.0291777.s001.zip › DiagramDataLiBowen/A01/A0103_26.png]

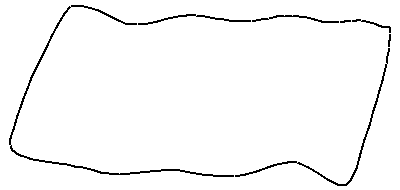

Supplement: S1 Data — (ZIP) [file pone.0291777.s001.zip › DiagramDataLiBowen/A01/A0103_27.png]

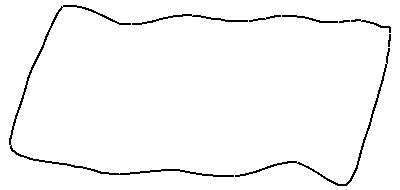

Supplement: S1 Data — (ZIP) [file pone.0291777.s001.zip › DiagramDataLiBowen/A01/A0103_28.png]

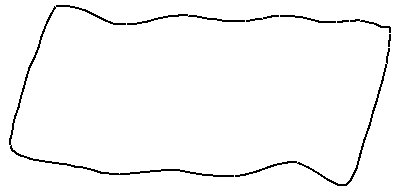

Supplement: S1 Data — (ZIP) [file pone.0291777.s001.zip › DiagramDataLiBowen/A01/A0103_29.png]

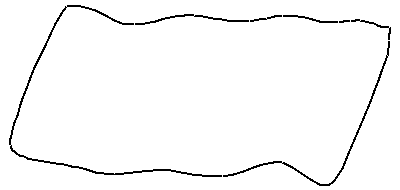

Supplement: S1 Data — (ZIP) [file pone.0291777.s001.zip › DiagramDataLiBowen/A01/A0103_3.png]
