# Supplementary figures and images for: Pumping machine fault diagnosis based on fused RDC-RBF (part 1 of 2)
Source: PLoS One. 2023 Sep 25;18(9):e0291777. doi: 10.1371/journal.pone.0291777 (PMC10519606; doi:10.1371/journal.pone.0291777)

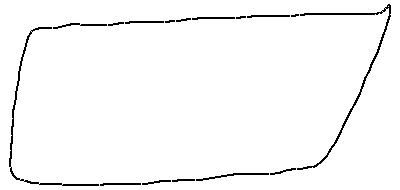

Supplement: S1 Data — (ZIP) [file pone.0291777.s001.zip › DiagramDataLiBowen/A01/A01_136214_0.png]

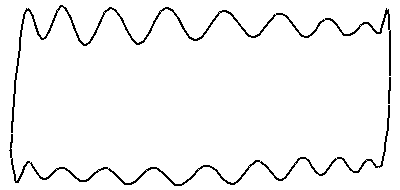

Supplement: S1 Data — (ZIP) [file pone.0291777.s001.zip › DiagramDataLiBowen/A01/A01_136214_1.png]

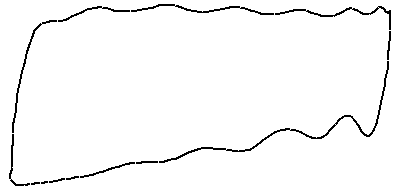

Supplement: S1 Data — (ZIP) [file pone.0291777.s001.zip › DiagramDataLiBowen/A01/A01_136214_10.png]

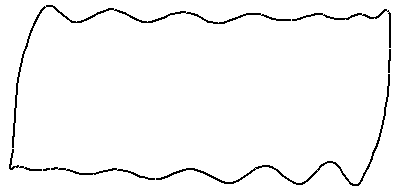

Supplement: S1 Data — (ZIP) [file pone.0291777.s001.zip › DiagramDataLiBowen/A01/A01_136214_11.png]

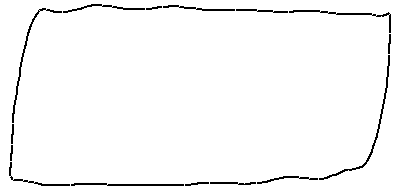

Supplement: S1 Data — (ZIP) [file pone.0291777.s001.zip › DiagramDataLiBowen/A01/A01_136214_12.png]

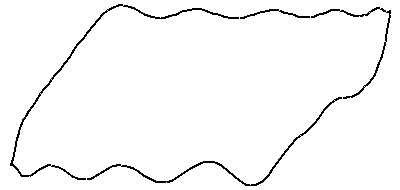

Supplement: S1 Data — (ZIP) [file pone.0291777.s001.zip › DiagramDataLiBowen/A01/A01_136214_13.png]

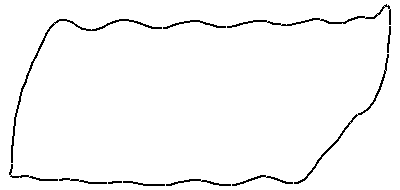

Supplement: S1 Data — (ZIP) [file pone.0291777.s001.zip › DiagramDataLiBowen/A01/A01_136214_14.png]

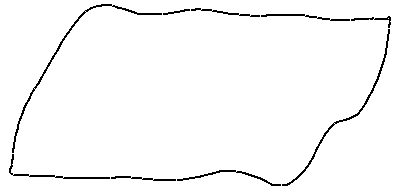

Supplement: S1 Data — (ZIP) [file pone.0291777.s001.zip › DiagramDataLiBowen/A01/A01_136214_15.png]

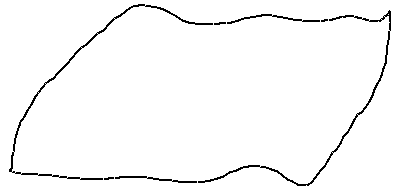

Supplement: S1 Data — (ZIP) [file pone.0291777.s001.zip › DiagramDataLiBowen/A01/A01_136214_16.png]

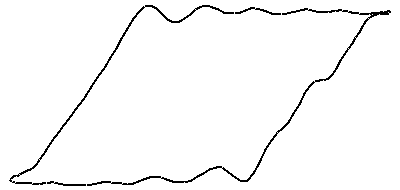

Supplement: S1 Data — (ZIP) [file pone.0291777.s001.zip › DiagramDataLiBowen/A01/A01_136214_17.png]

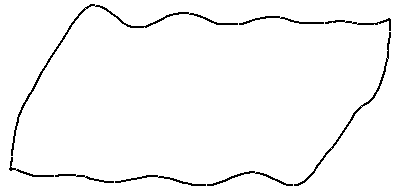

Supplement: S1 Data — (ZIP) [file pone.0291777.s001.zip › DiagramDataLiBowen/A01/A01_136214_18.png]

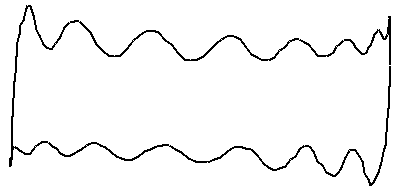

Supplement: S1 Data — (ZIP) [file pone.0291777.s001.zip › DiagramDataLiBowen/A01/A01_136214_19.png]

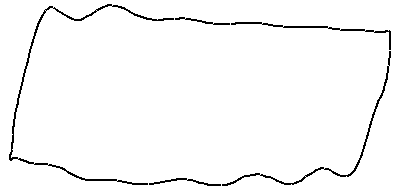

Supplement: S1 Data — (ZIP) [file pone.0291777.s001.zip › DiagramDataLiBowen/A01/A01_136214_2.png]

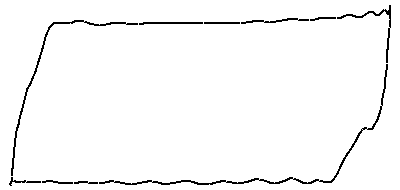

Supplement: S1 Data — (ZIP) [file pone.0291777.s001.zip › DiagramDataLiBowen/A01/A01_136214_20.png]

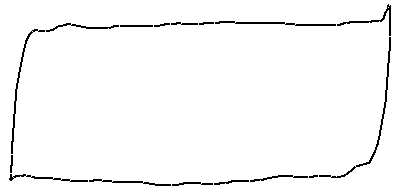

Supplement: S1 Data — (ZIP) [file pone.0291777.s001.zip › DiagramDataLiBowen/A01/A01_136214_21.png]

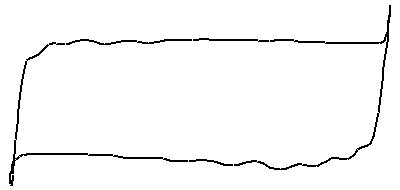

Supplement: S1 Data — (ZIP) [file pone.0291777.s001.zip › DiagramDataLiBowen/A01/A01_136214_22.png]

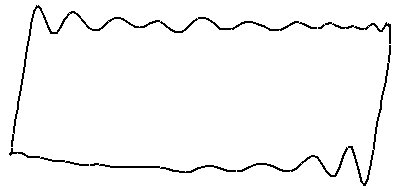

Supplement: S1 Data — (ZIP) [file pone.0291777.s001.zip › DiagramDataLiBowen/A01/A01_136214_23.png]

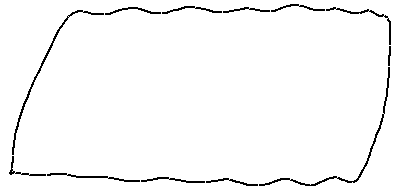

Supplement: S1 Data — (ZIP) [file pone.0291777.s001.zip › DiagramDataLiBowen/A01/A01_136214_3.png]

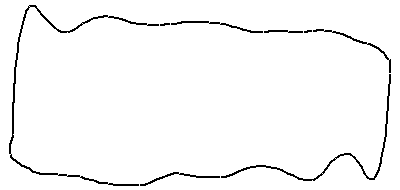

Supplement: S1 Data — (ZIP) [file pone.0291777.s001.zip › DiagramDataLiBowen/A01/A01_136214_4.png]

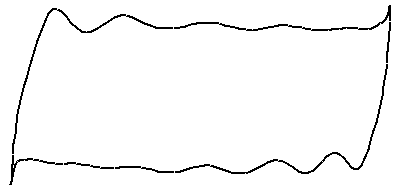

Supplement: S1 Data — (ZIP) [file pone.0291777.s001.zip › DiagramDataLiBowen/A01/A01_136214_5.png]

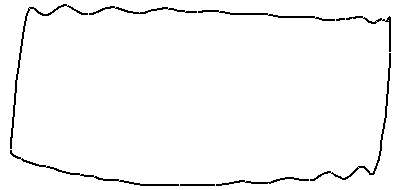

Supplement: S1 Data — (ZIP) [file pone.0291777.s001.zip › DiagramDataLiBowen/A01/A01_136214_6.png]

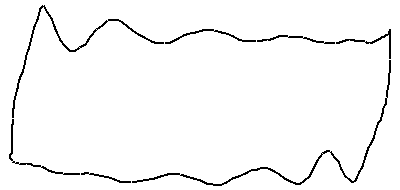

Supplement: S1 Data — (ZIP) [file pone.0291777.s001.zip › DiagramDataLiBowen/A01/A01_136214_7.png]

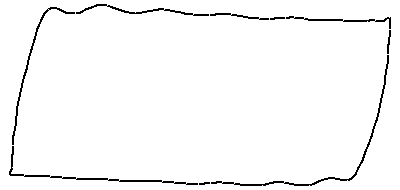

Supplement: S1 Data — (ZIP) [file pone.0291777.s001.zip › DiagramDataLiBowen/A01/A01_136214_8.png]

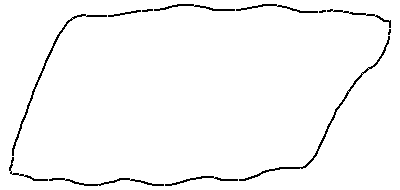

Supplement: S1 Data — (ZIP) [file pone.0291777.s001.zip › DiagramDataLiBowen/A01/A01_136214_9.png]

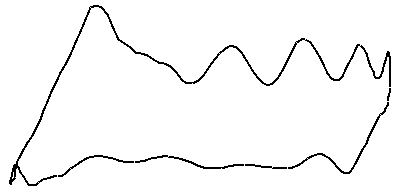

Supplement: S1 Data — (ZIP) [file pone.0291777.s001.zip › DiagramDataLiBowen/A01/A01_136959_0.png]

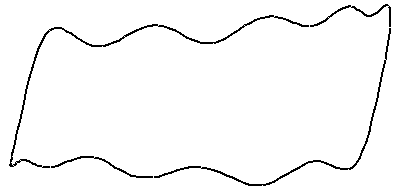

Supplement: S1 Data — (ZIP) [file pone.0291777.s001.zip › DiagramDataLiBowen/A01/A01_136959_1.png]

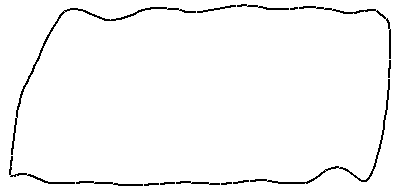

Supplement: S1 Data — (ZIP) [file pone.0291777.s001.zip › DiagramDataLiBowen/A01/A01_136959_10.png]

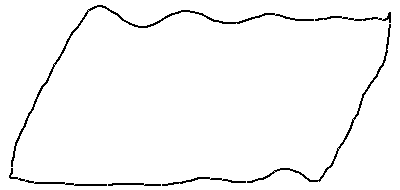

Supplement: S1 Data — (ZIP) [file pone.0291777.s001.zip › DiagramDataLiBowen/A01/A01_136959_11.png]

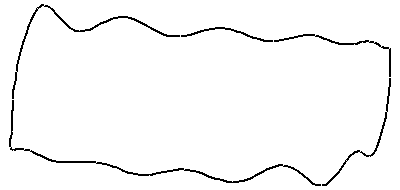

Supplement: S1 Data — (ZIP) [file pone.0291777.s001.zip › DiagramDataLiBowen/A01/A01_136959_12.png]

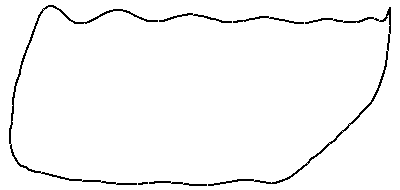

Supplement: S1 Data — (ZIP) [file pone.0291777.s001.zip › DiagramDataLiBowen/A01/A01_136959_13.png]

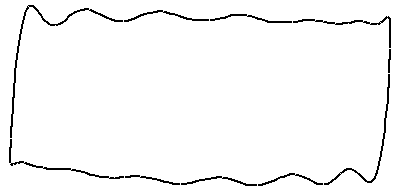

Supplement: S1 Data — (ZIP) [file pone.0291777.s001.zip › DiagramDataLiBowen/A01/A01_136959_14.png]

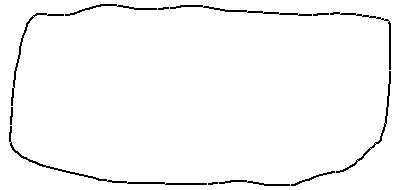

Supplement: S1 Data — (ZIP) [file pone.0291777.s001.zip › DiagramDataLiBowen/A01/A01_136959_15.png]

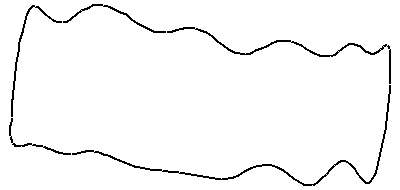

Supplement: S1 Data — (ZIP) [file pone.0291777.s001.zip › DiagramDataLiBowen/A01/A01_136959_2.png]

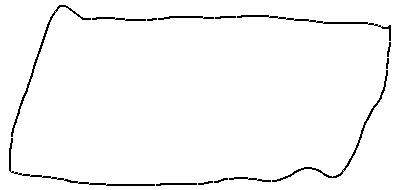

Supplement: S1 Data — (ZIP) [file pone.0291777.s001.zip › DiagramDataLiBowen/A01/A01_136959_3.png]

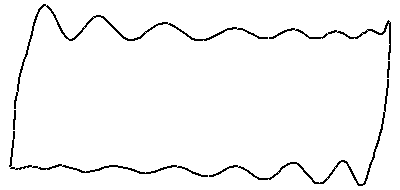

Supplement: S1 Data — (ZIP) [file pone.0291777.s001.zip › DiagramDataLiBowen/A01/A01_136959_4.png]

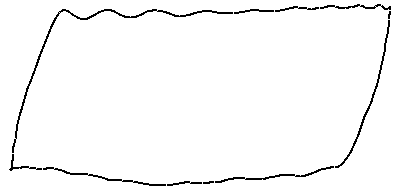

Supplement: S1 Data — (ZIP) [file pone.0291777.s001.zip › DiagramDataLiBowen/A01/A01_136959_5.png]

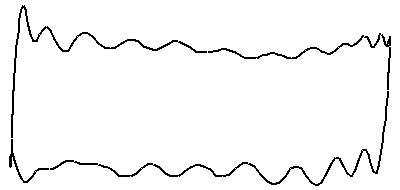

Supplement: S1 Data — (ZIP) [file pone.0291777.s001.zip › DiagramDataLiBowen/A01/A01_136959_6.png]

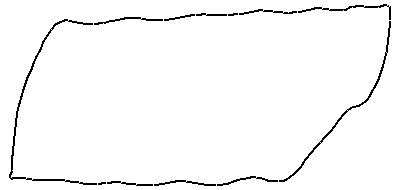

Supplement: S1 Data — (ZIP) [file pone.0291777.s001.zip › DiagramDataLiBowen/A01/A01_136959_7.png]

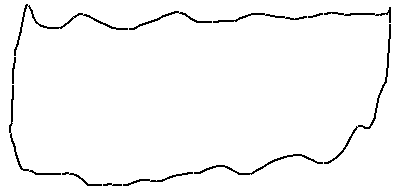

Supplement: S1 Data — (ZIP) [file pone.0291777.s001.zip › DiagramDataLiBowen/A01/A01_136959_8.png]

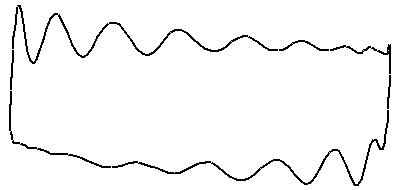

Supplement: S1 Data — (ZIP) [file pone.0291777.s001.zip › DiagramDataLiBowen/A01/A01_136959_9.png]

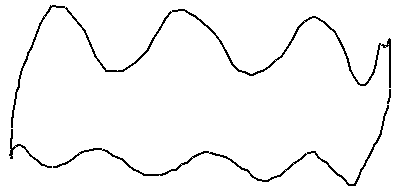

Supplement: S1 Data — (ZIP) [file pone.0291777.s001.zip › DiagramDataLiBowen/A01/A01_141421_30.png]

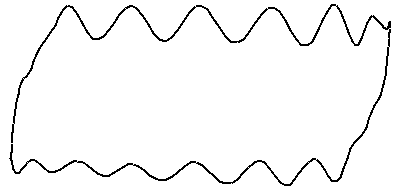

Supplement: S1 Data — (ZIP) [file pone.0291777.s001.zip › DiagramDataLiBowen/A01/A01_141421_31.png]

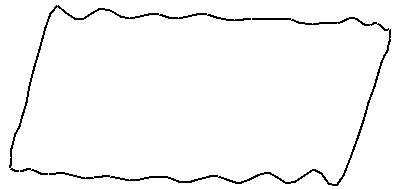

Supplement: S1 Data — (ZIP) [file pone.0291777.s001.zip › DiagramDataLiBowen/A01/A0101_1.png]

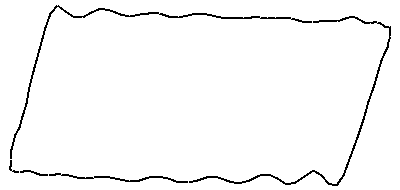

Supplement: S1 Data — (ZIP) [file pone.0291777.s001.zip › DiagramDataLiBowen/A01/A0101_10.png]

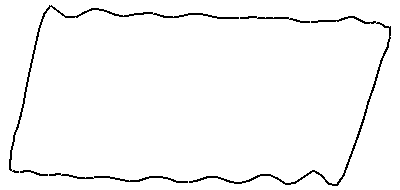

Supplement: S1 Data — (ZIP) [file pone.0291777.s001.zip › DiagramDataLiBowen/A01/A0101_11.png]

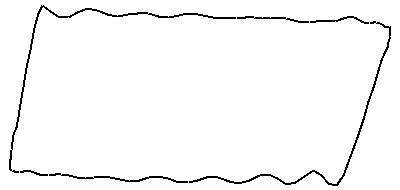

Supplement: S1 Data — (ZIP) [file pone.0291777.s001.zip › DiagramDataLiBowen/A01/A0101_12.png]

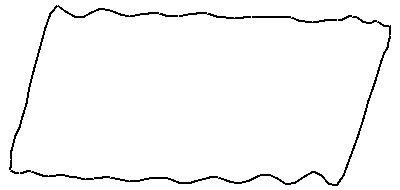

Supplement: S1 Data — (ZIP) [file pone.0291777.s001.zip › DiagramDataLiBowen/A01/A0101_13.png]

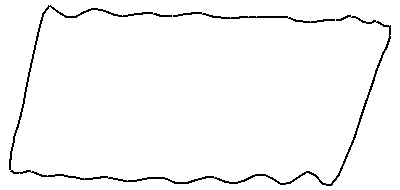

Supplement: S1 Data — (ZIP) [file pone.0291777.s001.zip › DiagramDataLiBowen/A01/A0101_14.png]

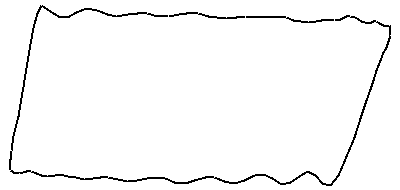

Supplement: S1 Data — (ZIP) [file pone.0291777.s001.zip › DiagramDataLiBowen/A01/A0101_15.png]

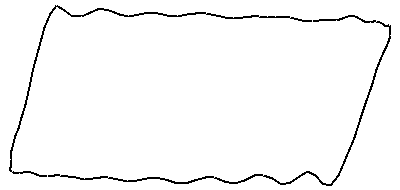

Supplement: S1 Data — (ZIP) [file pone.0291777.s001.zip › DiagramDataLiBowen/A01/A0101_16.png]

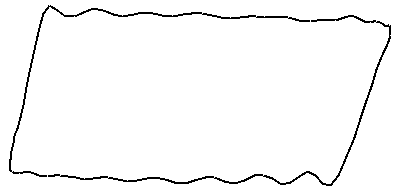

Supplement: S1 Data — (ZIP) [file pone.0291777.s001.zip › DiagramDataLiBowen/A01/A0101_17.png]

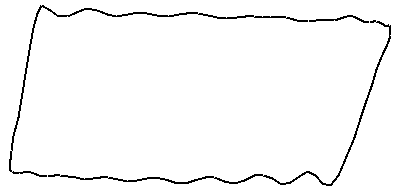

Supplement: S1 Data — (ZIP) [file pone.0291777.s001.zip › DiagramDataLiBowen/A01/A0101_18.png]

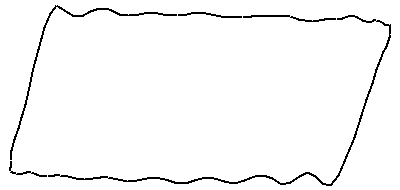

Supplement: S1 Data — (ZIP) [file pone.0291777.s001.zip › DiagramDataLiBowen/A01/A0101_19.png]

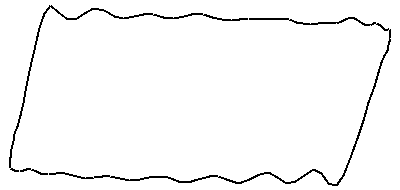

Supplement: S1 Data — (ZIP) [file pone.0291777.s001.zip › DiagramDataLiBowen/A01/A0101_2.png]

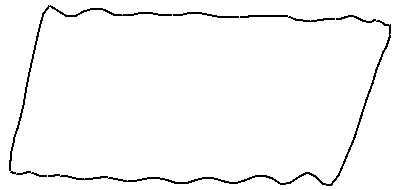

Supplement: S1 Data — (ZIP) [file pone.0291777.s001.zip › DiagramDataLiBowen/A01/A0101_20.png]

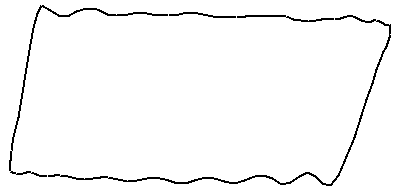

Supplement: S1 Data — (ZIP) [file pone.0291777.s001.zip › DiagramDataLiBowen/A01/A0101_21.png]

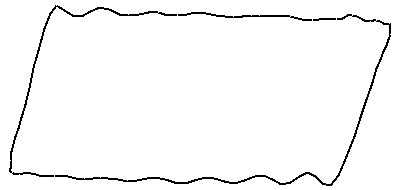

Supplement: S1 Data — (ZIP) [file pone.0291777.s001.zip › DiagramDataLiBowen/A01/A0101_22.png]

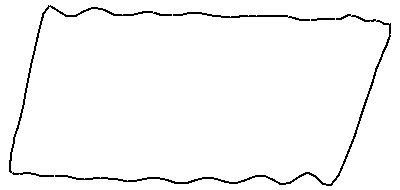

Supplement: S1 Data — (ZIP) [file pone.0291777.s001.zip › DiagramDataLiBowen/A01/A0101_23.png]

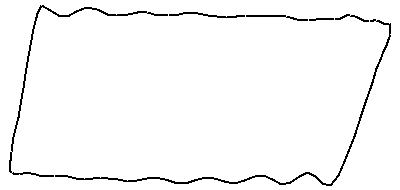

Supplement: S1 Data — (ZIP) [file pone.0291777.s001.zip › DiagramDataLiBowen/A01/A0101_24.png]

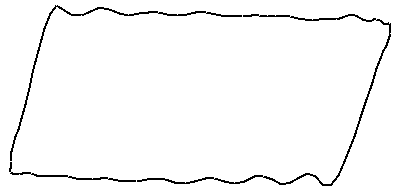

Supplement: S1 Data — (ZIP) [file pone.0291777.s001.zip › DiagramDataLiBowen/A01/A0101_25.png]

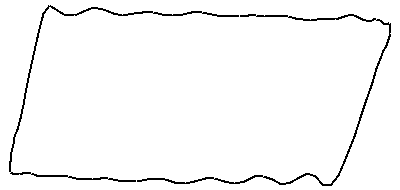

Supplement: S1 Data — (ZIP) [file pone.0291777.s001.zip › DiagramDataLiBowen/A01/A0101_26.png]

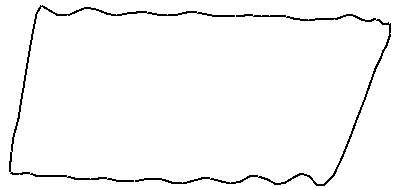

Supplement: S1 Data — (ZIP) [file pone.0291777.s001.zip › DiagramDataLiBowen/A01/A0101_27.png]

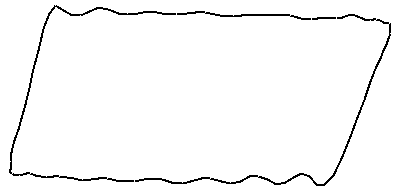

Supplement: S1 Data — (ZIP) [file pone.0291777.s001.zip › DiagramDataLiBowen/A01/A0101_28.png]

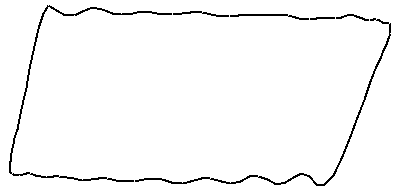

Supplement: S1 Data — (ZIP) [file pone.0291777.s001.zip › DiagramDataLiBowen/A01/A0101_29.png]

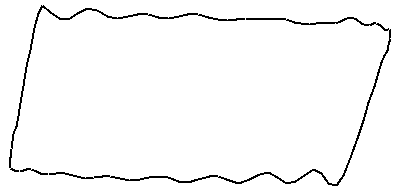

Supplement: S1 Data — (ZIP) [file pone.0291777.s001.zip › DiagramDataLiBowen/A01/A0101_3.png]

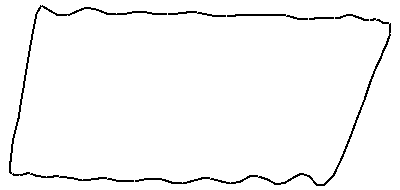

Supplement: S1 Data — (ZIP) [file pone.0291777.s001.zip › DiagramDataLiBowen/A01/A0101_30.png]

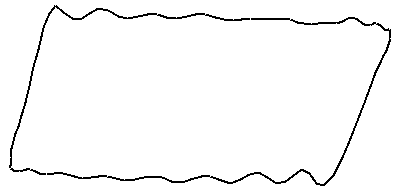

Supplement: S1 Data — (ZIP) [file pone.0291777.s001.zip › DiagramDataLiBowen/A01/A0101_31.png]

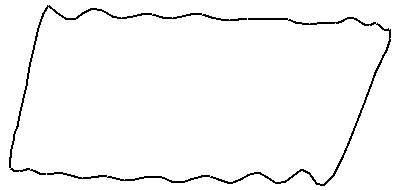

Supplement: S1 Data — (ZIP) [file pone.0291777.s001.zip › DiagramDataLiBowen/A01/A0101_32.png]

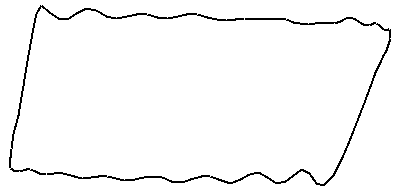

Supplement: S1 Data — (ZIP) [file pone.0291777.s001.zip › DiagramDataLiBowen/A01/A0101_33.png]

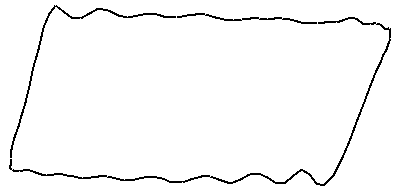

Supplement: S1 Data — (ZIP) [file pone.0291777.s001.zip › DiagramDataLiBowen/A01/A0101_34.png]

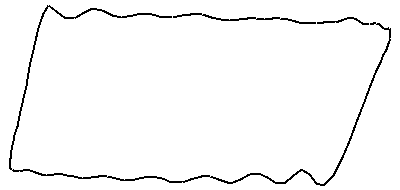

Supplement: S1 Data — (ZIP) [file pone.0291777.s001.zip › DiagramDataLiBowen/A01/A0101_35.png]

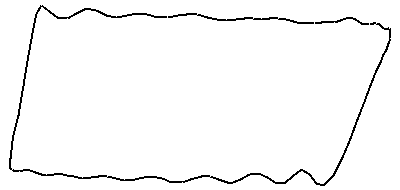

Supplement: S1 Data — (ZIP) [file pone.0291777.s001.zip › DiagramDataLiBowen/A01/A0101_36.png]

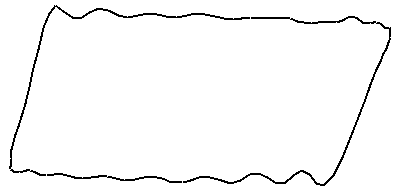

Supplement: S1 Data — (ZIP) [file pone.0291777.s001.zip › DiagramDataLiBowen/A01/A0101_37.png]

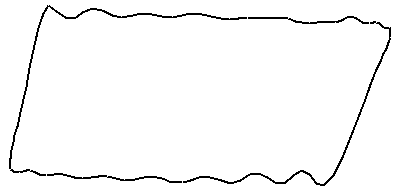

Supplement: S1 Data — (ZIP) [file pone.0291777.s001.zip › DiagramDataLiBowen/A01/A0101_38.png]

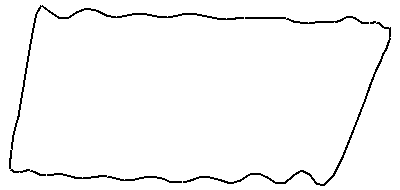

Supplement: S1 Data — (ZIP) [file pone.0291777.s001.zip › DiagramDataLiBowen/A01/A0101_39.png]

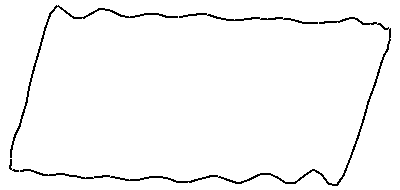

Supplement: S1 Data — (ZIP) [file pone.0291777.s001.zip › DiagramDataLiBowen/A01/A0101_4.png]

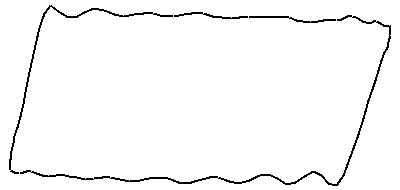

Supplement: S1 Data — (ZIP) [file pone.0291777.s001.zip › DiagramDataLiBowen/A01/A0101_44.png]

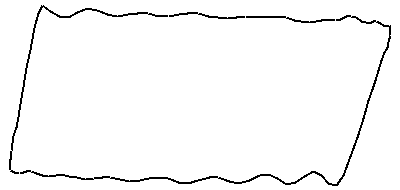

Supplement: S1 Data — (ZIP) [file pone.0291777.s001.zip › DiagramDataLiBowen/A01/A0101_45.png]

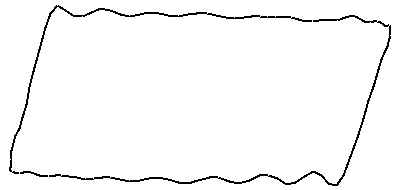

Supplement: S1 Data — (ZIP) [file pone.0291777.s001.zip › DiagramDataLiBowen/A01/A0101_46.png]

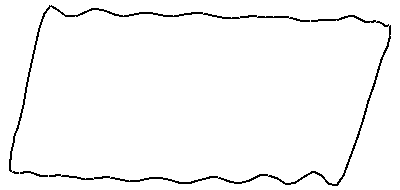

Supplement: S1 Data — (ZIP) [file pone.0291777.s001.zip › DiagramDataLiBowen/A01/A0101_47.png]

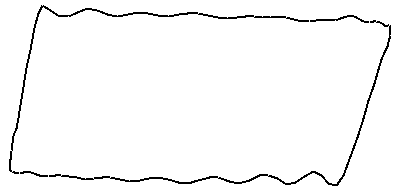

Supplement: S1 Data — (ZIP) [file pone.0291777.s001.zip › DiagramDataLiBowen/A01/A0101_48.png]

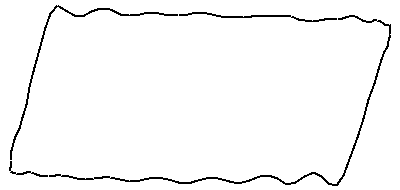

Supplement: S1 Data — (ZIP) [file pone.0291777.s001.zip › DiagramDataLiBowen/A01/A0101_49.png]

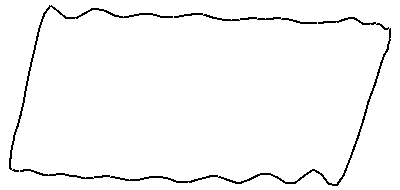

Supplement: S1 Data — (ZIP) [file pone.0291777.s001.zip › DiagramDataLiBowen/A01/A0101_5.png]

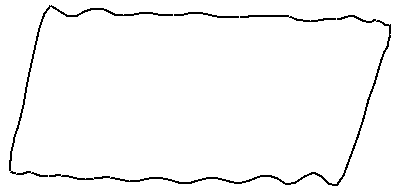

Supplement: S1 Data — (ZIP) [file pone.0291777.s001.zip › DiagramDataLiBowen/A01/A0101_50.png]

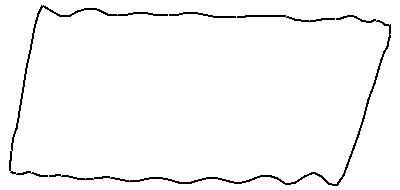

Supplement: S1 Data — (ZIP) [file pone.0291777.s001.zip › DiagramDataLiBowen/A01/A0101_51.png]

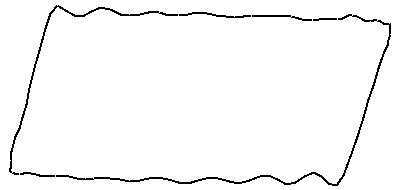

Supplement: S1 Data — (ZIP) [file pone.0291777.s001.zip › DiagramDataLiBowen/A01/A0101_52.png]

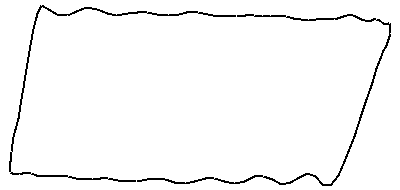

Supplement: S1 Data — (ZIP) [file pone.0291777.s001.zip › DiagramDataLiBowen/A01/A0101_57.png]

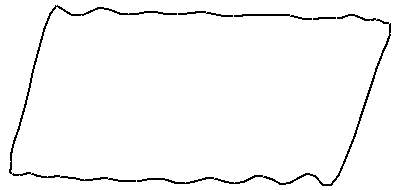

Supplement: S1 Data — (ZIP) [file pone.0291777.s001.zip › DiagramDataLiBowen/A01/A0101_58.png]

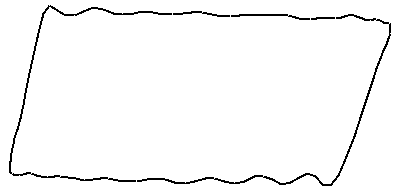

Supplement: S1 Data — (ZIP) [file pone.0291777.s001.zip › DiagramDataLiBowen/A01/A0101_59.png]

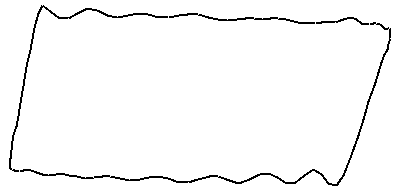

Supplement: S1 Data — (ZIP) [file pone.0291777.s001.zip › DiagramDataLiBowen/A01/A0101_6.png]

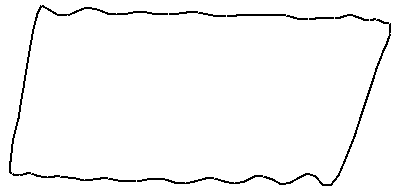

Supplement: S1 Data — (ZIP) [file pone.0291777.s001.zip › DiagramDataLiBowen/A01/A0101_60.png]

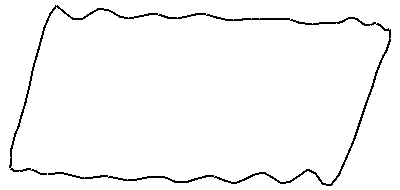

Supplement: S1 Data — (ZIP) [file pone.0291777.s001.zip › DiagramDataLiBowen/A01/A0101_61.png]

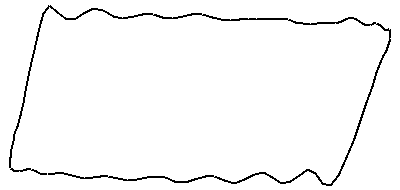

Supplement: S1 Data — (ZIP) [file pone.0291777.s001.zip › DiagramDataLiBowen/A01/A0101_62.png]

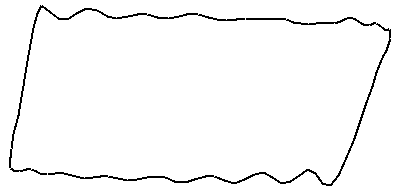

Supplement: S1 Data — (ZIP) [file pone.0291777.s001.zip › DiagramDataLiBowen/A01/A0101_63.png]

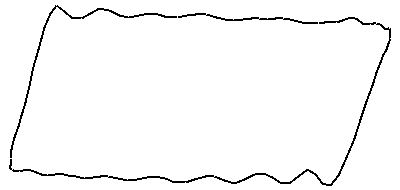

Supplement: S1 Data — (ZIP) [file pone.0291777.s001.zip › DiagramDataLiBowen/A01/A0101_64.png]

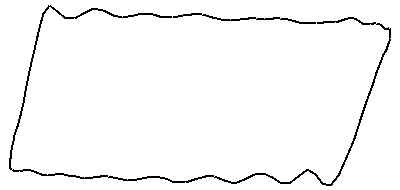

Supplement: S1 Data — (ZIP) [file pone.0291777.s001.zip › DiagramDataLiBowen/A01/A0101_65.png]

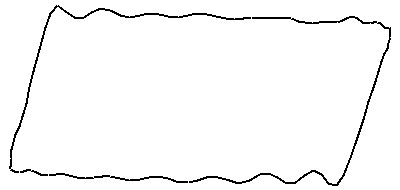

Supplement: S1 Data — (ZIP) [file pone.0291777.s001.zip › DiagramDataLiBowen/A01/A0101_7.png]

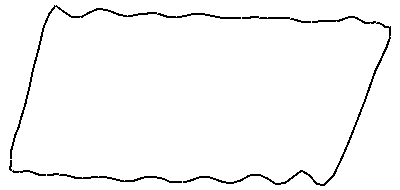

Supplement: S1 Data — (ZIP) [file pone.0291777.s001.zip › DiagramDataLiBowen/A01/A0101_70.png]

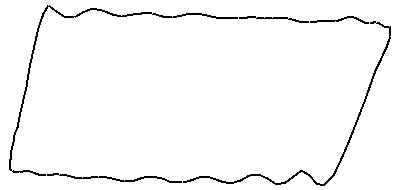

Supplement: S1 Data — (ZIP) [file pone.0291777.s001.zip › DiagramDataLiBowen/A01/A0101_71.png]

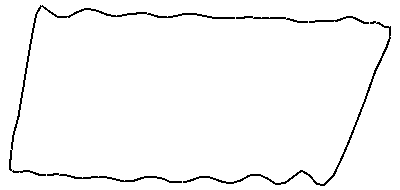

Supplement: S1 Data — (ZIP) [file pone.0291777.s001.zip › DiagramDataLiBowen/A01/A0101_72.png]
